# A phase I/IIa study of auceliciclib in patients with advanced solid tumours and in combination with temozolomide in patients with recurrent/relapsed high-grade glioma

**DOI:** 10.1016/j.esmoop.2025.106035

**Published:** 2026-01-24

**Authors:** T. Teo, J. Karanjia, P. Wabnitz, G. Kichenadasse, H.K. Gan, A. Cooper, D. Fuller, B. Noll, Y. Zhou, L. Wei, H. Wang, J. Liu, X. Zhou, K. Wang, S. Wang

**Affiliations:** 1 Aucentra Therapeutics Pty. Ltd., Adelaide, Australia; 2 clinPHARMA, St Andrew’s Hospital, Adelaide, Australia; 3 Southern Oncology Clinical Research Unit, Level 2 Flinders Private Hospital, Bedford Park, Australia; 4 Austin Hospital, Heidelberg, Australia; 5 Sydney Southwest Private Hospital, Liverpool, Australia; 6 Drug Discovery and Development, Clinical and Health Sciences, University of South Australia, Adelaide, Australia; 7 Changzhou Qianhong Biopharma Co. Ltd., Jiangsu, China; 8 Changzhou LeSun Pharmaceuticals Ltd., Jiangsu, China

**Keywords:** auceliciclib, phase I and IIa trial, advanced solid tumour, high-grade glioma, pharmacokinetics, safety

## Abstract

**Background:**

This first-in-human phase I/IIa study evaluated auceliciclib, a second-generation, highly selective cyclin-dependent kinase 4/6 (CDK4/6) inhibitor with potent antitumour activity, high brain penetration, and a wide preclinical therapeutic index. The trial assessed safety, tolerability, pharmacokinetics, and preliminary efficacy of auceliciclib as monotherapy in advanced solid tumours (phase I) and in combination with temozolomide for recurrent/relapsed high-grade glioma (phase IIa).

**Methods:**

This open-label study enrolled patients with advanced solid tumours or recurrent/relapsed high-grade glioma progressing after standard therapies. Dose escalation deployed accelerated titration followed by a 3 + 3 design. Phase I evaluated auceliciclib monotherapy (50-350 mg once daily; 175-500 mg twice daily). Phase IIa assessed auceliciclib (100-150 mg once daily; 100-500 mg twice daily) plus temozolomide (100 mg once daily). Regimens followed 21-day (once daily) or 28-day (twice daily) schedules per 28-day cycle.

**Results:**

Thirty-seven patients (20 in phase I; 17 in phase IIa) were treated. No dose-limiting toxicities were observed. Grade ≥3 auceliciclib-related TEAEs were infrequent (5.0% in phase I; 5.9% in phase IIa), with fatigue (40.5%), nausea (40.5%), vomiting (24.3%), and diarrhoea (21.6%) being the most common events. Pharmacokinetic analysis showed dose-dependent exposure, with twice-daily dosing yielding higher systemic levels, and a prolonged half-life at higher doses. Among 33 assessable patients, 14 achieved stable disease, including three high-grade glioma patients with disease control ≥24 weeks.

**Conclusions:**

Auceliciclib was well tolerated, and demonstrated dose-dependent pharmacokinetics and preliminary clinical activity in heavily pretreated patients. Recommended phase II doses are 500 mg twice daily (monotherapy) and 300 mg twice daily with 100 mg temozolomide once daily (combination therapy). These findings support further clinical development of auceliciclib for high-grade glioma and other malignancies.

## Highlights

• Auceliciclib is a second-generation, highly selective CDK4/6 inhibitor.
• It demonstrates minimal haematological toxicity—a key differentiator from currently approved CDK4/6 inhibitors.
• It shows early signs of clinical benefit when combined with temozolomide in the treatment of glioblastoma.
• Favourable tolerability and pharmacokinetic profile support its potential for broader therapeutic applications.

## Graphical abstract

**Figure:**
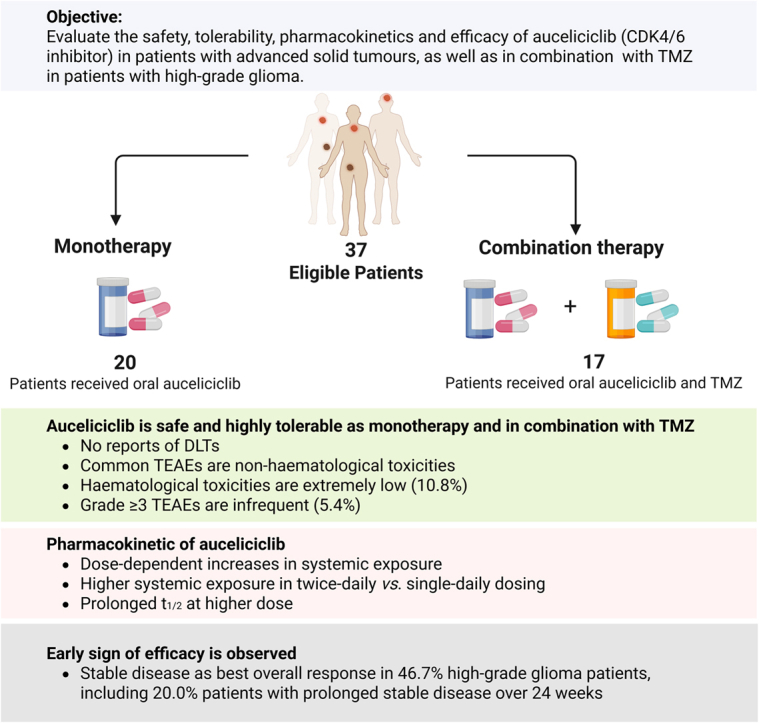

## Introduction

Advanced solid malignancies, particularly in the metastatic setting, pose a persistent therapeutic challenge. Despite incremental improvements in overall survival (OS) with contemporary systemic regimens, metastatic breast, colorectal, lung, ovarian, pancreatic, and primary central nervous system (CNS) cancers generally remain incurable, with limited gains in median survival. Standard-of-care (SOC) strategies integrate chemotherapy, targeted and endocrine therapies, surgery, radiotherapy, immunotherapy, and palliative care. Nevertheless, many patients progress through available options, highlighting an ongoing need for novel agents and combinations capable of improving outcomes in refractory metastatic disease.

The cyclin D1-cyclin-dependent kinase 4/6 (CDK4/6)-INK4-Rb axis is central to cell cycle control, and its dysregulation is a hallmark of oncogenesis. Genomic and epigenetic alterations, including *CDK4/6* amplification, *CDKN2A/B* deletion (encoding p16^INK4A^ and p15^INK4B^), *RB1* inactivation, and cyclin D1 overexpression, are common in aggressive malignancies. For instance, loss of p16^INK4A^ expression has been linked to unfavourable clinical outcomes and metastatic dissemination in locally advanced prostate cancer,^1^ and cyclin D1 overexpression correlates with shorter OS and progression-free survival (PFS) in advanced serous ovarian cancer.^2^, ^3^, ^4^ In colorectal cancer, overexpression of CDK4 and p16^INK4A^ is associated with accelerated proliferation, advanced tumour stage, and lymph node metastasis, respectively.^5^^,^^6^ These features highlight CDK4/6 as a significant therapeutic target and support biomarkers such as Rb proficiency and low p16^INK4A^ expression as potential predictors of CDK4/6 inhibitor response.

The Food and Drug Administration-approved CDK4/6 inhibitors, including palbociclib, ribociclib, and abemaciclib, have transformed the management of hormone receptor (HR)-positive/human epidermal growth factor receptor 2 (HER2)-negative advanced breast cancer by significantly improving PFS and/or OS when combined with endocrine therapy.^7^, ^8^, ^9^ The emergence of endocrine resistance, however, remains a considerable challenge in this setting, contributing to an increased risk of metastatic spread, including to the CNS. Approximately 10%-30% of patients with breast cancer develop brain metastases,^10^^,^^11^ which are associated with substantial morbidity and mortality.^12^ This unmet need has prompted evaluation of CDK4/6 inhibitors for enhancing intracranial disease control in both brain metastases and primary brain tumours. In a phase II trial of abemaciclib in HR-positive/HER2-negative metastatic breast cancer with brain metastases, abemaciclib demonstrated CNS penetration and modest intracranial activity, achieving an intracranial objective response rate of 5.2% and a clinical benefit rate of 24%, although the primary endpoint was not met.^13^^,^^14^ These findings support the mechanistic rationale for CDK4/6 inhibition in CNS malignancies, while highlighting current therapeutic limitations.

Glioblastoma (GBM), the most prevalent and aggressive high-grade malignant primary brain tumour, remains associated with a median survival of ∼15 months despite SOC comprising maximal resection, radiotherapy, and temozolomide (TMZ).^15^^,^^16^ TMZ, an oral alkylating agent that methylates guanine residues, improves survival versus radiotherapy alone but is limited by intrinsic and acquired resistance, including O^6^-methylguanine-DNA-methyltransferase (MGMT) promoter methylation status and mismatch repair proficiency.^17^ Following progression on SOC, most patients develop recurrent GBM (rGBM), for which no universally accepted SOC exists. Salvage strategies (e.g. re-operation, re-irradiation, nitrosoureas, bevacizumab-based regimens, targeted therapies) yield poor outcomes, with reported median OS ranging from 2.9 to 18.3 months and median PFS typically <6 months across randomised, controlled trials.^18^

Molecular analyses reveal that GBM is frequently characterised by dysregulation of the Rb pathway, with alterations in the cyclin D1-CDK4/6-INK4-Rb axis in ∼77% of cases,^19^ including *CDKN2A/B* deletions (up to 55%), *CDK4* amplification (14%), and *RB1* deletion (3%). These aberrations are associated with poor prognosis and support targeting CDK4/6 in GBM. Early clinical studies of CDK4/6 inhibitors in rGBM demonstrated CNS penetration and target engagement but only modest clinical benefit. A phase II trial of palbociclib in *RB1*-proficient rGBM reported median PFS and OS of 5.14 and 15.4 weeks, respectively, and was terminated early for futility.^20^ A phase 0 trial of ribociclib showed pharmacologically active intratumoural concentrations with median PFS of 9.7 weeks but limited clinical efficacy.^21^^,^^22^ Similarly, a pilot study of abemaciclib plus bevacizumab in biomarker-selected rGBM achieved median PFS and OS of 14 and 20 weeks, respectively, without benefit relative to historical bevacizumab monotherapy outcomes.^23^ Collectively, these data underscore the need for more effective CDK4/6-directed strategies.

The clinical utility of current CDK4/6 inhibitors is further constrained by dose-limiting toxicities (DLTs), largely attributed to off-target kinase inhibition.^24^ Palbociclib and ribociclib necessitate intermittent dosing to manage myelosuppression,^25^^,^^26^ while the more CDK4-selective abemaciclib (CDK4 : CDK6, 1 : 5) causes less haematological toxicity but dose-limiting gastrointestinal adverse events (AEs).^24^^,^^27^ These safety limitations, along with emerging resistance, have driven the pursuit of next-generation CDK4/6 inhibitors with enhanced kinase selectivity, improved tolerability, and reliable blood–brain barrier penetration, particularly for treatment-resistant CNS malignancies and other advanced cancers.

Auceliciclib (ulecaciclib, AU3-14) is a next-generation CDK4/6 inhibitor with marked CDK4 selectivity (CDK4/cyclin D1 *K*_i_ = 0.2 nM; CDK6/cyclin D3 *K*_i_ = 3 nM; CDK4 : CDK6 ≈ 1 : 15).^28^ Preclinical studies demonstrate potent antitumour activity with a wide therapeutic index across multiple *in vitro* and *in vivo* models, primarily via Rb-dependent cytostasis. In a U87 GBM xenograft model, auceliciclib combined with TMZ reversed acquired TMZ resistance and enhanced antitumour efficacy. Non-clinical pharmacokinetics (PK) and toxicology studies showed dose-dependent exposure and a manageable toxicity profile, with the absence of irreversible end-organ toxicity.^28^ These data supported initiation of a first-in-human phase I/IIa trial of auceliciclib in advanced solid malignancies and in combination with TMZ for high-grade glioma, including GBM (ATTACK-1).

## Methods

### Study design and procedures

This open-label, phase I/IIa study assessed the safety, tolerability, and PK of auceliciclib as monotherapy in locally advanced/metastatic solid tumours (phase I) and in combination with TMZ in recurrent/progressive high-grade glioma (phase IIa). Auceliciclib was administered once daily (od; 50-350 mg) on a 21-day schedule or twice daily (b.i.d.; 100-500 mg) on a 28-day schedule, with TMZ (100 mg od) added in phase IIa. Dose escalation deployed an accelerated titration followed by a 3 + 3 design, with DLTs assessed during cycle 1 to determine the maximum tolerated dose (MTD) and recommended phase II dose (RP2D). Safety assessments included physical examinations, vital signs, laboratory parameters, electrocardiograms, and AEs (CTCAE v5.0). Tumour response was assessed every two cycles (RECIST v1.1 for solid tumours and RANO criteria for brain tumours). Plasma auceliciclib concentrations were quantified by liquid chromatography–mass spectrometry (LC–MS)/MS, and PK parameters were derived by noncompartmental analysis (Phoenix WinNonlin v8.3). Additional methodological details are provided in the Supplementary Methods, available at https://doi.org/10.1016/j.esmoop.2025.106035.

### Ethics statement

This study followed the Declaration of Helsinki, ICH-GCP E6 (R2) guidelines, and the National Health and Medical Research Council’s National Statement on Ethical Conduct in Human Research (Clinical Trial Registry ID: ACTRN12621000479808). Protocol and amendments received institutional ethics approval. All participants provided written informed consent before enrolment.

## Results

### Enrolment, patient disposition, and disease characteristics

Between 2 June 2021 and 9 January 2024, a total of 45 patients were screened across three clinical sites in Australia, of whom 37 met the eligibility criteria and were enrolled in the study. In phase I, patients (*n* = 20) received auceliciclib doses at 50 mg od (*n* = 1), 100 mg od (*n* = 1), 150 mg od (*n* = 4), 250 mg od (*n* = 3), 350 mg od (*n* = 3), 175 mg b.i.d. (*n* = 3), 250 mg b.i.d. (*n* = 3) or 500 mg b.i.d. (*n* = 2). In phase IIa, patients (*n* = 17) were tested with auceliciclib doses at 100 mg od (*n* = 1), 150 mg od (*n* = 1), 100 mg b.i.d. (*n* = 5), 150 mg b.i.d. (*n* = 3), 300 mg b.i.d. (*n* = 3) or 500 mg b.i.d. (*n* = 4), in addition to 100 mg of TMZ od.

Baseline patient demographics and disease characteristics are summarised in Table 1. A total of 20 patients with locally advanced (25%) or metastatic (75%) solid tumours were enrolled in phase I. Diagnoses included brain cancer (20%, encompassing GBM, anaplastic oligodendroglioma, and meningioma), pancreatic cancer (15%), liver cancer (10%), endometrial cancer (10%), colorectal (10%) or rectosigmoid adenocarcinoma (5%), low-grade serous ovarian cancer (LGSOC; 5%), non-small-cell lung cancer (NSCLC; 5%), cervical cancer (5%), pleural mesothelioma (5%), chordoma (5%), and small-cell neuroendocrine carcinoma (5%). Seventeen patients with high-grade glioma were enrolled in phase IIa.

**Table 1:** Patient demographics and disease characteristics at baseline

Characteristic	Phase I (*n* = 20)	Phase IIa (*n* = 17)
Median age, years (range)	62.5 (27-82)	58.0 (38-70)
Sex, *n* (%)		
Male	9 (45)	14 (82.4)
Female	11 (55)	3 (17.6)
Race, *n* (%)		
White	19 (95)	15 (88.2)
Asian	1 (5)	2 (11.8)
Tumour type, *n* (%)		
Brain cancer	4 (20)	17 (100)
Pancreatic cancer	3 (15)	—
Colorectal adenocarcinoma	2 (10)	—
Liver cancer	2 (10)	—
Endometrial cancer	2 (10)	—
Non-small-cell lung cancer	1 (5)	—
Low-grade serous ovarian cancer	1 (5)	—
Cervical cancer	1 (5)	—
Pleural mesothelioma	1 (5)	—
Chordoma	1 (5)	—
Small-cell neuroendocrine carcinoma	1 (5)	—
Rectosigmoid cancer	1 (5)	—
Extent of disease, *n* (%)		
Locally advanced	5 (25)	15 (88.2)
Metastatic	15 (75)	2 (11.8)
Prior cancer therapy, *n* (%)	20 (100)	17 (100)
Prior antineoplastic surgery, *n* (%)	18 (90)	17 (100)
Prior radiotherapy, *n* (%)	13 (65)	17 (100)
Time from end of radiotherapy to first dose, median (range), months	16.4 (16.1-100)^a^	17.0 (0.95-63.8)
≥6 months, *n* (%)^b^	4 (100)^a^	16 (94.1)
Re-irradiation, *n* (%)	—	2 (11.8)^c^

a Only included patients with brain tumours who completed Stupp-protocol radiotherapy (*n* = 4).

b Patients enrolled ≥6 months after radiotherapy.

c Re-irradiation was administered 13.8 and 2.3 months before the first auceliciclib dose in the two respective patients.

All patients had received prior anticancer therapy. In phase I, 65.0% had prior radiotherapy and 90.0% had prior antineoplastic surgery; in phase IIa, all patients had received both (Table 1). A small subset of patients with brain tumours (6% per subgroup in phase IIa) entered the study within 6 months of radiotherapy or following recent re-irradiation (i.e. <6 months). These cases (flagged in Supplementary Table S1, available at https://doi.org/10.1016/j.esmoop.2025.106035) may have treatment-related imaging changes that confound assessment stability. By study completion (9 January 2024), all patients had discontinued treatment, primarily due to disease progression (70% in phase I; 64.7% in phase IIa), followed by patient withdrawal (10% in phase I; 11.8% in phase IIa), AEs (10% in phase I; 11.8% in phase IIa), other reasons (5% in phase I; 11.8% in phase IIa), and death (5% in phase I) (Supplementary Table S2, available at https://doi.org/10.1016/j.esmoop.2025.106035). Notably, two (11.8%) patients with high-grade glioma in phase IIa continued auceliciclib/TMZ combination under the Special Access Scheme (SAS; Supplementary Table S1, available at https://doi.org/10.1016/j.esmoop.2025.106035).

### Safety and tolerability

#### Phase I

A total of 131 treatment-emergent AEs (TEAEs) were reported in 95% (*n* = 19) of patients enrolled in phase I, with 38 of these TEAEs in 70% (*n* = 14) of patients deemed related to auceliciclib (Supplementary Table S3, available at https://doi.org/10.1016/j.esmoop.2025.106035). The most common all-grade TEAEs (≥10%) included non-haematological events such as nausea (30.0%), diarrhoea (25.0%), vomiting (15.0%), fatigue (15.0%), and headache (15.0%) (Table 2). Anaemia (10.0%) was the only haematological AE, occuring in two patients. All common TEAEs were grade ≤2 in severity, self-limiting, and manageable with concomitant medications.

**Table 2:** Adverse events (≥5% all grades) deemed related to auceliciclib in phase I

Adverse event, *n* (%) M^a^	Grade	50 mg od (*n* = 1)	100 mg od (*n* = 1)	150 mg od (*n* = 4)	250 mg od (*n* = 3)	350 mg od (*n* = 3)	175 mg b.i.d. (*n* = 3)	250 mg b.i.d. (*n* = 3)	500 mg b.i.d. (*n* = 2)	All^b^ (*n* = 20)
Nausea	All	0	0	1 (25) 1	1 (33.3) 1	1 (33.3) 1	1 (33.3) 1	1 (33.3) 1	1 (50) 3	6 (30) 8
	Grade 3	0	0	0	0	0	0	0	0	0
Diarrhoea	All	0	0	0	0	1 (33.3) 1	0	2 (66.7) 2	2 (100) 3	5 (25) 6
	Grade 3	0	0	0	0	0	0	0	0	0
Vomiting	All	0	0	1 (25) 1	0	0	0	1 (33.3) 1	1 (50) 1	3 (15) 3
	Grade 3	0	0	0	0	0	0	0	0	0
Fatigue	All	0	0	0	0	1 (33.3) 1	0	1 (33.3) 3	1 (50) 1	3 (15) 5
	Grade 3	0	0	0	0	0	0	0	0	0
Headache	All	0	0	0	2 (66.7) 2	0	0	1 (33.3) 1	0	3 (15) 3
	Grade 3	0	0	0	0	0	0	0	0	0
Anaemia	All	1 (100) 1	0	1 (25) 1	0	0	0	0	0	2 (10) 2
	Grade 3	0	0	0	0	0	0	0	0	0
Investigations—other: liver function test increased^c^	All	1 (100) 2	0	0	0	0	0	0	0	1 (5) 2
	Grade 3	1 (100) 1	0	0	0	0	0	0	0	1 (5) 1
Abdominal distension	All	0	0	0	0	1 (33.3) 1	0	0	0	1 (5) 1
	Grade 3	0	0	0	0	0	0	0	0	0
Constipation	All	0	0	1 (25) 1	0	0	0	0	0	1 (5) 1
	Grade 3	0	0	0	0	0	0	0	0	0
Dry skin	All	0	0	0	0	0	0	0	1 (50) 1	1 (5) 1
	Grade 3	0	0	0	0	0	0	0	0	0
Dry mouth	All	0	0	0	0	0	0	1 (33.3) 1	0	1 (5) 1
	Grade 3	0	0	0	0	0	0	0	0	0
Belching	All	0	0	0	0	0	0	1 (33.3) 1	0	1 (5) 1
	Grade 3	0	0	0	0	0	0	0	0	0
General disorders and administration site conditions—other: mucositis	All	0	0	0	0	0	0	1 (33.3) 1	0	1 (5) 1
	Grade 3	0	0	0	0	0	0	0	0	0
Dysgeusia	All	0	0	0	0	0	0	0	1 (50) 1	1 (5) 1
	Grade 3	0	0	0	0	0	0	0	0	0
ALT increased	All	0	0	0	0	0	0	0	1 (50) 1	1 (5) 1
	Grade 3	0	0	0	0	0	0	0	0	0
AST increased	All	0	0	0	0	0	0	0	1 (50) 1	1 (5) 1
	Grade 3	0	0	0	0	0	0	0	0	0

ALT, alanine aminotransferase; AST, aspartate aminotransferase; b.i.d., twice daily; *n*, sample size; od, once daily.

a The number of patients is counted once for the highest relationship to auceliciclib for a given adverse event in patient count (*n*). Occurrences are counted each time in events (M).

b No more than grade 3 events reported.

c The term liver function test increased was applied to collectively describe the increases in alanine aminotransferase, alkaline phosphatase, and gamma-glutamyl transferase observed in the patient.

No life-threatening TEAEs, treatment withdrawal, dose reductions, or deaths related to TEAEs were observed at doses up to 1000 mg daily of auceliciclib (Supplementary Table S3, available at https://doi.org/10.1016/j.esmoop.2025.106035). Additionally, no serious AEs were attributed to auceliciclib.

#### Phase IIa

All patients (*n* = 17) in phase IIa experienced a total of 139 TEAEs, with 48 and 47 of these TEAEs in 94.1% (*n* = 16) of patients deemed related to auceliciclib and TMZ, respectively (Supplementary Table S3, available at https://doi.org/10.1016/j.esmoop.2025.106035). The most common all-grade TEAEs (≥10%) included fatigue (70.6%) and gastrointestinal events such as nausea (52.9%), vomiting (35.3%), and diarrhoea (17.6%) (Table 3). Haematological AEs, including decreased platelet and lymphocyte counts, were reported in two (11.8%) patients. These AEs were managed with supportive therapy and/or dose adjustments/interruptions. Two (11.8%) patients required dose interruptions, and two (11.8%) discontinued treatment due to TEAEs. Notably, the only two grade 3 AEs, platelet count decreased and lymphocyte count decreased, occurred in the same patient who was enrolled in the highest dose cohort (500 mg b.i.d.).

**Table 3:** Adverse events (≥5% all grades) deemed related to study treatment (auceliciclib and TMZ) in phase IIa

Adverse event, *n* (%) M^a^	Grade	100 mg od (*n* = 1)	150 mg od (*n* = 1)	100 mg b.i.d. (*n* = 5)	150 mg b.i.d. (*n* = 3)	300 mg b.i.d. (*n* = 3)	500 mg b.i.d. (*n* = 4)	All (*n* = 17)^b^
Fatigue	All	1 (100) 1	1 (100) 1	3 (60.0) 4	2 (66.7) 3	3 (100) 3	2 (50) 3	12 (70.6) 15
	Grade 3	0	0	0	0	0	0	0
Nausea	All	1 (100) 1	0	1 (20) 1	1 (33.3) 1	3 (100) 3^c^	3 (75) 3	9 (52.9) 9
	Grade 3	0	0	0	0	0	0	0
Vomiting	All	1 (100) 1	0	0	2 (66.7) 2	1 (33.3) 1	2 (50) 2	6 (35.3) 6
	Grade 3	0	0	0	0	0	0	0
Diarrhoea	All	0	0	0	0	2 (66.7) 3	1 (25) 2	3 (17.6) 5
	Grade 3	0	0	0	0	0	0	0
Platelet count decreased	All	0	0	1 (20) 1	0	0	1 (25) 2	2 (11.8) 3
	Grade 3	0	0	0	0	0	1 (25) 1	1 (5.9) 1
Abdominal pain	All	0	0	0	0	0	1 (25) 1	1 (5.9) 1
	Grade 3	0	0	0	0	0	0	0
Dysgeusia	All	0	0	0	0	0	1 (25) 1	1 (5.9) 1
	Grade 3	0	0	0	0	0	0	0
ALT increased	All	0	0	0	0	0	1 (25) 1	1 (5.9) 1
	Grade 3	0	0	0	0	0	0	0
Gastrointestinal disorders—other: geographic tongue	All	0	0	0	1 (33.3) 1	0	0	1 (5.9) 1
	Grade 3	0	0	0	0	0	0	0
Rash pustular	All	0	0	0	1 (33.3) 1	0	0	1 (5.9) 1
	Grade 3	0	0	0	0	0	0	0
Upper respiratory infection	All	0	0	1 (20.0) 1	0	0	0	1 (5.9) 1
	Grade 3	0	0	0	0	0	0	0
Anorexia	All	0	0	0	0	1 (33.3) 1	0	1 (5.9) 1
	Grade 3	0	0	0	0	0	0	0
Skin and subcutaneous tissue disorders—other: dermatitis	All	0	0	0	1 (33.3) 1	0	0	1 (5.9) 1
	Grade 3	0	0	0	0	0	0	0
Bursitis	All	0	0	1 (20) 1^d^	0	0	0	1 (5.9) 1
	Grade 3	0	0	0	0	0	0	0
Lipase increased	All	0	0	0	1 (33.3) 1	0	0	1 (5.9) 1
	Grade 3	0	0	0	0	0	0	0
Lymphocyte count decreased	All	0	0	0	0	0	1 (25) 1^e^	1 (5.9) 1
	Grade 3	0	0	0	0	0	1 (25) 1	1 (5.9) 1

ALT, alanine aminotransferase; b.i.d., twice daily; *n,* sample size; od, once daily; TMZ, temozolomide.

a The number of patients is counted once for the highest relationship to auceliciclib for a given adverse event in patient count (*n*). Occurrences are counted each time in events (M).

b No more than grade 3 events reported.

c One of the adverse events was not related to TMZ.

d Adverse event related to auceliciclib only.

e Adverse event related to TMZ only.

No life-threatening TEAEs or deaths related to TEAEs were observed at doses up to 1000 mg daily of auceliciclib combined with 100 mg of TMZ (Supplementary Table S3, available at https://doi.org/10.1016/j.esmoop.2025.106035).

#### Cardiac safety

Preclinical data suggested a potential risk of corrected QT interval (QTc) prolongation at high doses of auceliciclib, based on observed increases in heart rate of monkeys. No clinically significant arrhythmias or QT interval corrected for heart rate using Fridericia’s formula (QTcF) intervals >450 ms or increases >30 ms from baseline were observed, however, except for one patient (100 mg b.i.d. cohort in phase IIa) with a QTcF interval of 451 ms in cycle 14.

#### Dose-limiting toxicity and maximum tolerated dose

No DLTs were observed in any dose cohort during the DLT evaluation period, and the MTD was not reached.

### Efficacy

Some 89.2% (*n* = 33) of patients were assessable for efficacy assessments including Eastern Cooperative Oncology Group (ECOG) and Neurologic Assessment in Neuro-Oncology (NANO) (patients with brain tumours only) performance status, alongside radiographic response (Supplementary Table S4, available at https://doi.org/10.1016/j.esmoop.2025.106035). No patients experienced a substantial deterioration in ECOG status (defined as >2 point increase) (Supplementary Table S5, available at https://doi.org/10.1016/j.esmoop.2025.106035). Transient 2 point ECOG increases from baseline were observed in 21.2% of patients during post-dose evaluations. One patient with a baseline ECOG of 2 progressed to a maximum score of 4, attributed to disease progression. NANO domain scores demonstrated minimal variability from baseline, with most patients exhibiting stability or a 1 point increase (Supplementary Table S6, available at https://doi.org/10.1016/j.esmoop.2025.106035). No dose-dependent or treatment-related patterns in ECOG/NANO changes were identified, suggesting preserved neurologic and functional status across dosing cohorts.

Per RECIST v1.1 or RANO criteria, stable disease (SD) was the best overall response in 38.9% of patients in phase I (Table 4), encompassing pancreatic (*n* = 2), endometrial (*n* = 1), liver (*n* = 1), LGSOC (*n* = 1), NSCLC (*n* = 1), and meningioma (*n* = 1) tumours (Supplementary Table S1, available at https://doi.org/10.1016/j.esmoop.2025.106035). The median PFS was 7.57 weeks [95% confidence interval (CI) 6.29-11 weeks]. In phase IIa (*n* = 15), 46.7% of high-grade glioma (predominantly GBM) patients achieved SD, with prolonged SD ≥24 weeks in 20% patients (Table 4). The median PFS extended to 10 weeks (95% CI 7.14-43.43 weeks), indicative of potential clinical benefit.

**Table 4:** Tumour response and efficacy results per RECIST v1.1 or RANO criteria

	50 mg od (*n* = 1)	100 mg od (*n* = 1)	150 mg od (*n* = 4)	250 mg od (*n* = 3)	350 mg od (*n* = 2)	175 mg b.i.d. (*n* = 2)	250 mg b.i.d. (*n* = 3)	500 mg b.i.d. (*n* = 2)	Phase I (*n* = 18)
Best overall response^a^, *n* (%)									
Complete response	0	0	0	0	0	0	0	0	0
Partial response	0	0	0	0	0	0	0	0	0
Stable disease	1 (100)	0	0	1 (33.3)	1 (50)	0	2 (66.7)	2 (100)	7 (38.9)
≥24 weeks	0	0	0	0	0	0	0	0	0
<24 weeks	1 (100)	0	0	1 (33.3)	1 (50)	0	2 (66.7)	2 (100)	7 (38.9)
Progressive disease	0	1 (100)	4 (100)	2 (66.7)	1 (50)	2 (100)	1 (33.3)	0	11 (61.1)
Disease control rate^b^, *n* (%)	1 (100)	0	0	1 (33.3)	1 (50.0)	0	2 (66.7)	2 (100.0)	7 (38.9)
Median PFS (95% CI)^c^, weeks	7.71 (NA)	6.29 (NA)	6.71 (1.43-NA)	8.00 (7.29-NA)	6.36 (5.57-NA)	7.43 (3.86-NA)	17.71 (10.14-NA)	11.71 (NA)	7.57 (6.29-11.00)

	100 mg od (*n* = 1)	150 mg od (*n* = 1)	100 mg b.i.d. (*n* = 5)	150 mg b.i.d. (*n* = 3)	300 mg b.i.d. (*n* = 3)	500 mg b.i.d. (*n* = 2)	Phase IIa (*n* = 15)	All (*n* = 33)
Best overall response^a^, *n* (%)								
Complete response	0	0	0	0	0	0	0	0
Partial response	0	0	0	0	0	0	0	0
Stable disease	0	1 (100)	3 (60)	1 (33.3)	1 (33.3)	1 (50.0)	7 (46.7)	14 (42.4)
≥24 weeks	0	1 (100)	1 (20)	1 (33.3)	0	0	3 (20)	3 (9.1)
<24 weeks	0	0	2 (40)	0	1 (33.3)	1 (50.0)	4 (26.7)	11 (33.3)
Progressive disease	1 (100)	0	2 (40)	2 (66.7)	2 (66.7)	1 (50.0)	8 (53.3)	19 (57.6)
Disease control rate^b^, *n* (%)	0	1 (100)	3 (60)	1 (33.3)	1 (33.3)	1 (50.0)	7 (46.7)	14 (42.4)
Median PFS (95% CI)^c^, weeks	7.29 (NA)	43.43 (NA)	12.29 (7.43-NA)	7.86 (3.00-NA)	7.14 (4.43-NA)	— (8.29-NA)	10.00 (7.14-43.43)	8.00 (7.29-11.00)

b.i.d., twice daily; CI, confidence interval; *n,* sample size; NA, not available; PFS, progression-free survival; od, once daily; RANO, response assessment in neuro-oncology; RECIST, response evaluation criteria in solid tumours.

a The best overall response was defined as the best response a patient had following start of dosing, up to and including RECIST/RANO progression/death, or the last evaluable assessment in the absence of RECIST/RANO progression/death.

b Disease control was defined as a best overall response of complete response or partial response or having stable disease from the first dose of auceliciclib.

c PFS is defined as the time from first dose until the date of first documented disease progression by RECIST/RANO criteria or death (by any cause in the absence of disease progression), regardless of whether the patient withdraws from study therapy or receives another anticancer therapy before progression. Patients who have not progressed or died at the time of analysis are censored at the time of the latest date of assessment from their last evaluable disease assessment. If the patient progresses or dies after two or more consecutive missed visits, however, the patient is censored at the time of the latest evaluable disease assessment before the two missed visits. Median PFS and 95% CI were calculated using the Kaplan–Meier technique.

Within the monotherapy cohorts receiving 50-350 mg od, median PFS remained relatively consistent (6.29-8.00 weeks). In contrast, higher b.i.d. doses of 250 mg and 500 mg were associated with longer median PFS of 17.71 and 11.71 weeks, respectively. No clear dose-PFS relationship was observed in phase IIa. These findings suggest a potential early efficacy signal with b.i.d. monotherapy dosing; however, the limited sample sizes warrant cautious interpretation and confirmation with larger cohorts.

### Pharmacokinetics

**Figure 1:**
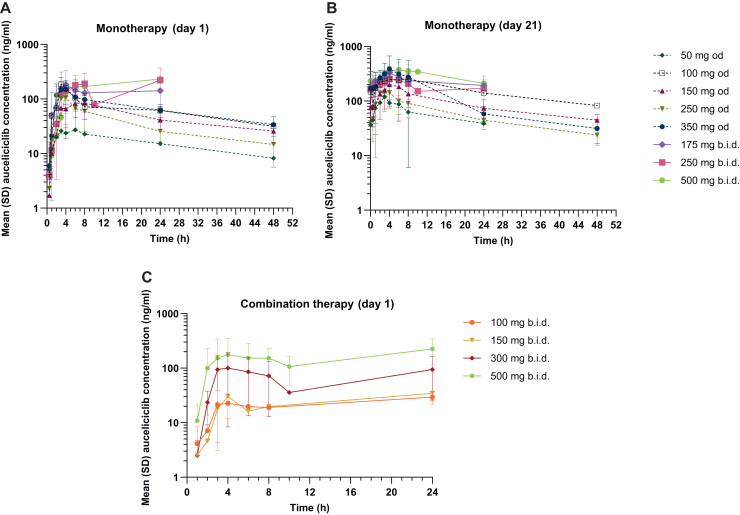
**Plasma concentration profiles of auceliciclib following oral administration.** Mean (± standard deviation) plasma concentration-time curves of auceliciclib following total daily dosing across a dose range of 50 to 1000 mg on cycle 1 (A) day 1 and (B) day 21, and of auceliciclib in combination with 100 mg TMZ, with twice daily doses ranging from 100 to 500 mg on cycle 1 (C) day 1. b.i.d., twice daily; od, once daily; SD, standard deviation.

PK analysis of auceliciclib following single-dose administration (Figure 1A and Supplementary Table S7, available at https://doi.org/10.1016/j.esmoop.2025.106035) revealed dose-dependent increases in maximum plasma concentration (C_max_) and area under the concentration-time curve (AUC). C_max_ ranged from 27.2 ng/ml (50 mg od) to 232 ng/ml (500 mg b.i.d.), with corresponding AUC values increasing from 747 to ∼4000-4180 h × ng/ml at the highest doses. Nonetheless, substantial inter-subject variability in AUC was noted within certain dose groups, particularly the od regimens (Supplementary Figure S1, available at https://doi.org/10.1016/j.esmoop.2025.106035). While the time to C_max_ (t_max_) varied (e.g. 6 h at 50 mg od and around 24 h at 500 mg b.i.d.), the terminal elimination half-life (t_1/2_) remained relatively stable across dose groups (i.e. 20-32.7 h).

Repeated dosing further increased systemic exposure (Figure 1B and Supplementary Table S7, available at https://doi.org/10.1016/j.esmoop.2025.106035). For example, at 50 mg od, C_max_ increased from 27.2 ng/ml (single dose) to 120 ng/ml (repeated dose), and AUC_0-tlast_ rose from 747 to 1510 h × ng/ml. Similar or greater increases were observed at higher doses, such as 500 mg b.i.d. where C_max_ increased from 232 to 383 ng/ml and AUC_0-tlast_ from 4000 to 6760 h × ng/ml. T_max_ after repeated doses was relatively stable (3.07-7.6 h) with reduced inter-subject variability. The t_1/2_ remained comparable to single dosing (17.2-26.3 h). Repeated dosing was associated with decreased apparent clearance (CL/F) and apparent volume of distribution (V_z_/F). For example, at 50 mg od, CL/F decreased from 47.1 l/h (single dose) to 33.6 l/h (repeated dose), and V_z_/F from 1810 to 836 L. Overall, the data indicate a clear dose-dependent increase in C_max_ and AUC with higher doses resulting in substantially higher exposure and reduced CL/F. Significant inter-subject variability was observed in both single and repeated dose PK parameters, particularly at higher doses (Supplementary Figure S2, available at https://doi.org/10.1016/j.esmoop.2025.106035).

Co-administration of auceliciclib with TMZ also showed dose-dependent PK. C_max_ and AUC of auceliciclib increased with escalating doses (e.g. C_max_ = 12.3 ng/ml and AUC_0-tlast_ = 236 h × ng/ml for 100 mg od; C_max_ = 239 ng/ml and AUC_0-tlast_ = 3900 h × ng/ml for 500 mg b.i.d.) following single dose administration (Figure 1C, Supplementary Table S8 and Figures S3 and S4, available at https://doi.org/10.1016/j.esmoop.2025.106035). Limited repeated-dose data indicated further increases in exposure (e.g. C_max_ = 160 ng/ml and AUC_0-tlast_ = 1020 h × ng/ml for 300 mg b.i.d. to C_max_ = 292 ng/ml and AUC_0-tlast_ = 2210 h × ng/ml for 500 mg b.i.d.). Due to the limited sampling, analyses of t_1/2_, CL/F, and V_z_/F were not available. Overall, the PK profile of auceliciclib/TMZ combination exhibits dose-dependent PK, with significant increases in systemic exposure correlating with escalating doses. Repeated dosing of auceliciclib led to higher overall exposure compared with single-dose administration. Further comprehensive PK studies encompassing all relevant parameters will be necessary to optimise dosing strategies for this combination therapy.

## Discussion

This phase I/IIa trial provides the first clinical assessment of auceliciclib, a novel CDK4/6 inhibitor with preferential CDK4 selectivity and broad kinase specificity, evaluating its safety and tolerability as monotherapy in advanced solid tumours and in combination with TMZ in high-grade glioma, predominantly GBM. Auceliciclib demonstrated a favourable safety profile, with continuous oral dosing up to 1000 mg daily well tolerated and no MTD reached. This continuous administration distinguishes auceliciclib from intermittently scheduled CDK4/6 inhibitors, supporting the potential of auceliciclib for sustained therapeutic exposure.

In phase I, the most common TEAEs were gastrointestinal toxicities, including nausea (30.0%), diarrhoea (25.0%), and vomiting (15.0%), along with fatigue (15.0%) and headache (15.0%). Grade ≥3 TEAEs were infrequent (5.0%). Although gastrointestinal events increased with b.i.d. dose escalation, they were predominantly mild to moderate, manageable with supportive care, and self-limiting.

In phase IIa, the most frequent TEAEs were fatigue (70.6%), nausea (52.9%), vomiting (35.3%), and diarrhoea (17.6%). Grade ≥3 TEAEs occurred in 5.9% of patients. Treatment discontinuation due to toxicity was rare (two patients within the 500 mg b.i.d. cohort; Supplementary Table S9, available at https://doi.org/10.1016/j.esmoop.2025.106035): one for grade 2 nausea and fatigue (nausea resolved within 1 day of discontinuation and fatigue persisting at end-of-study) and one for grade 1 diarrhoea that began 6 days before the last dose and was improving with supportive medications at the end-of-study assessment. The rapid resolution or improvement of these events, together with the low discontinuation rate, indicates that auceliciclib-related toxicities were generally manageable and reversible.

Noteworthy, auceliciclib exhibited a relatively low incidence of haematological toxicities, despite such events being a well-established class effect of CDK4/6 inhibition. In pivotal trials of approved CDK4/6 inhibitors for metastatic breast cancer, palbociclib and ribociclib routinely induce grade ≥3 neutropenia in 50%-70% of treated patients,^29^, ^30^, ^31^ reflecting potent CDK6 inhibition and associated suppression of haematopoietic stem and progenitor cell proliferation.^32^^,^^33^ In contrast, auceliciclib demonstrated a markedly attenuated haematologic profile across early-phase studies: in phase I monotherapy, only two low-grade anaemia events (10.0%) were observed at lower dose levels (i.e. 50 mg od and 150 mg od). Similarly, in phase IIa, haematological events remained infrequent (11.8%; *n* = 2), including one case of severe lymphocyte depletion accompanied by a progressive decline in platelet counts, necessitating interruption of TMZ. These events were consistent with the known myelosuppressive effects of TMZ,^34^ which targets rapidly dividing haematopoietic stem cells (HSCs).^35^

Mechanistically, CDK6 plays a key role in the transcriptional regulation of HSC activation,^32^ where its inhibition has been linked to the development of anaemia and neutropenia.^36^ The infrequent and predominantly low-grade haematological toxicities associated with auceliciclib monotherapy, despite some CDK6 inhibition, align with its preclinical CDK4-selective profile.^28^ This selectivity likely mitigates bone marrow suppression and contributes to a differentiated safety profile compared with less selective CDK4/6 inhibitors.^24^

Preclinically identified concerns regarding QTc interval prolongation were not observed clinically. No patients exhibited QTcF increases >30 ms from baseline, indicating a low apparent risk of drug-induced arrhythmia at the studied doses. Nonetheless, given the precedent within the class,^25^^,^^26^^,^^37^ the effect of auceliciclib on cardiac safety will remain a focus of future clinical investigations.

PK analyses demonstrated dose-dependent increases in auceliciclib exposure (50-1000 mg daily), albeit with large interpatient variability. Twice daily dosing produced higher C_max_ and AUC_0-tlast_ than od dosing, supporting b.i.d. administration. Co-administration with 100 mg TMZ appeared to reduce auceliciclib C_max_ (292 versus 383 ng/ml) and AUC_0-tlast_ (2210 versus 6760 h × ng/ml) versus auceliciclib monotherapy at an equivalent dose (500 mg b.i.d.). Conversely, preclinical PK analyses indicated a significant increase in the CNS exposure of both agents in combination,^28^ suggesting altered plasma–brain distribution. Mechanistically, auceliciclib undergoes primary hepatic metabolism via CYP3A4, while TMZ is not hepatically metabolised,^34^ thereby reducing direct metabolic competition. Furthermore, the lack of marked differences in t_max_ and CL/F between the two 500 mg b.i.d. cohorts suggests that competition in absorption and clearance mechanisms is also unlikely. The limited sample sizes, however, particularly with some cohorts consisting of single subjects, contributed to high variability and potentially imprecise PK estimates. This inherent limitation reduces the statistical power and generalisability of the findings. Future studies with larger sample sizes, particularly at doses within and around the expansion range, are essential for more robust characterisation of auceliciclib PK alone and with TMZ.

Despite the absence of genetic stratification to predict treatment response, preliminary signs of clinical activity with auceliciclib were observed. In the phase I and phase IIa studies, 38.9% (*n* = 7) and 46.7% (*n* = 7) of assessable patients, respectively, achieved SD, including three high-grade glioma patients in phase IIa who maintained durable SD for ≥24 weeks. While cross-trial comparisons are inherently limited, this level of disease stabilisation appears comparable with historical experience from other CDK4/6 inhibitors. For instance, palbociclib and ribociclib demonstrated disease control rates (DCRs) of 35.0% and 32.6%, respectively, in heavily pretreated solid tumour populations,^26^^,^^38^ whereas abemaciclib produced SD in 16 of 29 assessable patients (55.2%) in the first-in-human dose-escalation study, with DCRs of 13%-27% in subsequent expansion cohorts across diverse tumour types, excluding populations with known preferential responses to abemaciclib.^27^ When compared with metronomic TMZ in heavily pretreated rGBM, which was associated with a DCR of 42.5% and substantial lymphocytopenia (35%, including grade ≥3 in 10%),^39^ the auceliciclib/TMZ combination demonstrated comparable disease control with a markedly more favourable haematological tolerability profile (11.8%).

Systemic options for rGBM are limited and generally confer modest benefit. Lomustine, a commonly used alkylating agent, yields median OS of 8.6-9.8 months and PFS of 1.5-2.7 months, with grade ≥3 thrombocytopenia in 13%-25% of patients.^40^, ^41^, ^42^ While regorafenib showed improved median OS versus lomustine in REGOMA (7.4 versus 5.6 months), the median PFS remained limited at 2.0-2.7 months, and the benefit was offset by an increased incidence of grade ≥3 non-haematological AEs.^41^^,^^43^ In this context, the disease control and durability observed with auceliciclib, together with its favourable haematological profile, appear clinically meaningful and support further evaluation in recurrent/relapsed high-grade glioma.

Preclinical PK assessments indicate that auceliciclib achieves therapeutically relevant CNS concentrations, exceeding the threshold required for CDK4 inhibition in intracranial tissue.^28^ This is likely facilitated by its low susceptibility to P-glycoprotein efflux. Although definitive confirmation of target engagement within intracranial tumours was not feasible, the clinical outcomes observed with auceliciclib/TMZ combination in high-grade glioma provide supportive evidence of CNS pharmacological activity. The MGMT promoter methylation status, a key predictor of TMZ sensitivity in high-grade glioma,^44^^,^^45^ did not appear to significantly influence sensitivity to the auceliciclib/TMZ combination among the 17 enrolled high-grade glioma patients, most of whom had progressed after multiple prior therapies. Of the six patients with confirmed unmethylated MGMT promoters (Supplementary Table S10, available at https://doi.org/10.1016/j.esmoop.2025.106035), 50% achieved SD. Three individual cases highlighted durable disease control in this refractory population. The first patient, who had previously progressed after four cycles of TMZ monotherapy, maintained SD for 11 cycles on the auceliciclib/TMZ combination, despite having an unknown MGMT status. The second patient, with a confirmed unmethylated MGMT promoter and early progression on TMZ (fewer than four cycles), achieved durable disease control for more than 14 trial cycles, and an additional seven cycles in the subsequent SAS. The third patient, harbouring MGMT promoter methylation but not assessable for radiographic response to prior TMZ after fewer than eight prior cycles of TMZ, remained progression-free for 11 trial cycles and continued in the SAS for more than 16 additional cycles. These clinical observations align with preclinical findings^28^ suggesting that auceliciclib may restore or enhance TMZ sensitivity irrespective of MGMT status.

Furthermore, a non-linear dose-response was observed in high-grade glioma, indicating that factors beyond dose intensity are likely to influence therapeutic effect. The mechanism by which CDK4/6 inhibition sensitises tumours to TMZ, particularly in an MGMT-independent manner, remains unclear. One plausible explanation is that CDK4/6 inhibition induces sustained G1 arrest and impairs DNA damage responses,^46^ thereby promoting accumulation of TMZ-induced DNA damage and/or replication stress on cell cycle re-entry.^47^ Inter-patient variability in tumour biology, drug metabolism, and PK, and the glioma microenvironment may further modulate this pharmacodynamic interaction and contribute to outcome variability.

Pharmacodynamic sampling was limited in this study; future trials could incorporate feasible surrogate tissues (e.g. circulating tumour DNA, peripheral blood mononuclear cells) to evaluate CDK4/6 target engagement (e.g. reductions in phosphorylated Rb) and DNA damage markers (e.g. induction of γH2AX and cleaved caspase 3). Retrospective correlative studies using archival tumour are also planned to interrogate molecular alterations within the cyclin D1-CDK4/6-INK4-Rb axis. Variability in *RB1* proficiency, *CDKN2A/B* deletion, or *CDK4*/*CCND1* amplification is likely to influence sensitivity to CDK4/6 inhibition and may have attenuated efficacy signals or masked intrinsic resistance mechanisms in this unselected, all-comer population. Identifying predictive biomarkers, such as intact *RB1* with preserved Rb expression, loss of inhibitory CDK regulators, or DNA mismatch repair status, may enable biomarker-driven patient selection in future expansion cohorts and subsequent trials, thereby supporting the development of a more personalised therapeutic strategy for advanced solid tumours, particularly high-grade glioma.

Based on cumulative safety, PK, and preliminary efficacy data, the recommended starting doses are 500 mg b.i.d. for auceliciclib monotherapy and 300 mg b.i.d. with 100 mg TMZ in 28-day cycles.

### Conclusions

This first-in-human phase I/IIa trial demonstrates that auceliciclib, a second-generation CDK4/6 inhibitor with preferential CDK4 targeting, is clinically well tolerated as monotherapy in advanced solid tumours and in combination with TMZ for high-grade glioma. Auceliciclib demonstrated low-risk, reversible haematological toxicity, a clinically significant dose-limiting AE of approved CDK4/6 inhibitors, while maintaining dose-dependent PK and evidence of preliminary clinical benefit, particularly in the phase IIa combination cohorts. These findings suggest that auceliciclib may not only improve therapeutic outcomes but also broaden the therapeutic indications for CDK4/6 inhibition by minimising toxicity across a wide therapeutic index. Given its promising safety and pharmacologic profile, further clinical investigation is warranted, ideally incorporating pharmacodynamic and biomarker-driven patient selection to define populations most likely to benefit.
