# Supplementary material for: A phase I/IIa study of auceliciclib in patients with advanced solid tumours and in combination with temozolomide in patients with recurrent/relapsed high-grade glioma
Source: ESMO Open. 2026 Jan 24;11(2):106035. doi: 10.1016/j.esmoop.2025.106035 (PMC12860713; doi:10.1016/j.esmoop.2025.106035)
Supplement: Supplementary Data [file mmc1.pdf]

A phase I/IIa study of auceliciclib in patients with advanced solid tumours and in combination with temozolomide in patients with recurrent/relapsed high-grade glioma

T. Teo[1], J. Karanjia[1], P. Wabnitz[2], G. Kichenadasse[3], H. K. Gan[4], A. Cooper[5], D. Fuller[1], B. Noll[6], Y. Zhou[7], L. Wei[7], H. Wang[8], J. Liu[8], X. Zhou[7], K. Wang[7] & S. Wang[1,6*]

[1]Aucentra Therapeutics Pty. Ltd., Adelaide; [2]clinPHARMA, St Andrew's Hospital, Adelaide; [3]Southern Oncology Clinical Research Unit, Level 2 Flinders Private Hospital, Bedford Park; [4]Austin Hospital, Heidelberg; [5]Sydney Southwest Private Hospital, Liverpool; [6]Drug Discovery and Development, Clinical and Health Sciences, University of South Australia, Adelaide, Australia; [7]Changzhou Qianhong Biopharm Co. Ltd., Jiangsu; [8]Changzhou LeSun Pharmaceuticals Ltd., Jiangsu, China.

*Correspondence to: Prof. Shudong Wang, Drug Discovery and Development, Clinical and Health Sciences, University of South Australia, Adelaide, SA 5000, Australia. Tel: +61 883022372 Email: shudong.wang@unisa.edu.au (S. Wang).

Supplementary Methods

Study Objectives

The primary objectives of this study were to assess the safety and tolerability of auceliciclib, determine dose-limiting toxicities (DLTs), and establish the maximum tolerated dose (MTD) and recommended phase II dose (RP2D). Secondary objectives included full pharmacokinetics (PK) characterisation following single and repeated dosing, as well as evaluation of preliminary anti-tumour activity.

The MTD was defined as the highest dose at which less than one-third of evaluable patients experienced DLTs. The RP2D was selected based on overall safety, tolerability, and PK considerations without exceeding the MTD.

Patients Eligibility

Adults (≥ 18 years) with histologically or cytologically confirmed locally advanced or metastatic cancers, recurrent or progressive high-grade gliomas (e.g. GBM) following standard-of-care (SOC) treatment were eligible. Patients must have had no other effective SOC therapies available. Any number of prior chemotherapies or radiotherapies was permitted. Eligibility criteria included an Eastern Cooperative Oncology Group (ECOG) performance status of 0 – 2, adequate organ function, a life expectancy of at least three months, and measurable disease per response evaluation criteria in solid tumours (RECIST) version 1.1 or response assessment in neuro-oncology (RANO) criteria. Prior cancer-directed therapies, including anti-tumour therapy (excluding TMZ), radiation, and investigational devices or agents, must have been discontinued at least 28 days before study entry. Reproductive health measures were mandatory for patients of childbearing potential.

Exclusion criteria included prior treatment with a CDK4/6 inhibitor, untreated or unstable primary CNS tumours or CNS metastases, active infections, recent major surgery, unresolved adverse effects from prior therapies, or concurrent use of strong inducers or inhibitors of cytochrome P450 3A (CYP3A).

Rationale of auceliciclib starting dose

The starting dose of auceliciclib was determined in accordance with relevant regulatory guidelines for non-clinical evaluation of anticancer agents.[1,2] In 28-day repeated dose toxicology studies, the highest non-severely toxic dose (HNSTD) in rats and monkeys was established as 100 and 75 mg/kg, respectively, which were translated to human equivalent doses of 16.2 and 24.3 mg/kg, respectively, using recommended allometric scaling factors. While a safety factor of six is generally acceptable, a more conservative approach was implemented for dose calculation due to the presence of residual pathological findings observed during recovery necropsy in the toxicology studies. Consequently, a starting dose of 50 mg was selected for this study, equivalent to 0.83 mg/kg for a 60 kg individual. This dose represents an estimated safety factor of approximately 20-fold relative to the HNSTD in the most sensitive species (i.e. the rat).

Rationale of hybrid dose-escalation study design

A hybrid dose-escalation approach, consisting of an initial accelerated titration phase followed by a conventional 3+3 escalation, was deployed to balance efficiency and patient safety in this first-in-human study. This strategy was informed by non-clinical data suggesting a wide therapeutic window, and by the methodological framework proposed by Simon *et al.*,[3] which aims to minimise patient exposure to sub-therapeutic doses while maintaining robust safety monitoring.

In the accelerated phase, dose escalation commenced with single-patient cohorts, with each patient monitored for DLTs or treatment-related Grade ≥2 adverse events during the cycle 1 day 1-28 DLT evaluation period. Escalation to the next dose level proceeded only if no such events were observed. If a patient in these accelerated cohorts experienced a Grade ≥2 event not clearly attributable to underlying

disease, other medical conditions, or concomitant medications or procedures during the DLT period, the cohort was expanded by enrolling two additional patients and applying standard 3+3 decision rules; if a DLT occurred, the cohort was further expanded to six patients. Beyond the second dose level, and irrespective of whether toxicity had been observed during the accelerated phase, all subsequent dose levels followed a conventional 3+3 design to ensure more comprehensive assessment of DLTs, safety, and PK.

Definition of DLT

DLT was defined as treatment-related grade 3/4 toxicities occurring during days 1-28 of cycle 1. Non-haematological toxicities, such as fatigue, weakness, nausea, vomiting, diarrhoea, or electrolyte abnormalities, were considered dose-limiting only if they persisted for more than two days despite optimal supportive care. Grade 3 elevations in aspartate aminotransferase, alanine aminotransferase, and/or total bilirubin lasting less than seven days, grade 3 thrombocytopenia without bleeding, grade 3 lymphopenia, and grade 3 neutropenia without fever or requiring growth factor support for <7 days were not classified as DLTs. Additionally, any treatment-emergent adverse event (TEAE) during the DLT period, regardless of grade, was considered a DLT if it resulted in treatment discontinuation, dose reduction, or administration of less than 75% of planned doses, provided the TEAE was not attributable to the patient's underlying disease, other medical conditions, or concomitant therapies or procedures.

Pharmacokinetic assessments

Plasma auceliciclib concentrations were quantified using a validated liquid chromatography–mass spectrometry (LC–MS/MS) assay with a lower limit of quantification of 2.0 ng/mL. In phase I, PK samples were collected during cycle 1 at the following time points: day 1 (predose and at 1, 2, 3, 4, 6, 8, and 10 hours post-dose), day 2 (predose), day 3 (predose), day 8 (predose), day 15 (predose), day 21 (predose and at 1, 2, 3, 4, 6, 8, and 10 hours post-dose), day 22 (predose), and day 23 (predose). During cycle 2, PK sampling was conducted on day 1 (predose), day 15 (predose), and day 22 (predose). From cycle 3 onwards, PK samples were obtained on day 1 (predose) and day 22 (predose). In phase IIa, prior to protocol amendment, repeated-dose PK samples (i.e. day 21) were not collected during cycle 1. Furthermore, the PK sampling schedule for phase IIa excluded sample collection on day 3, day 8, day 22, and day 23.

Statistical analyses

Safety and efficacy analyses included all patients who received ⩾1 dose of auceliciclib. DLT evaluation for MTD determination included patients completing the DLT period. PK analyses included patients with sufficient data for reliable parameter estimation.

Safety analyses included descriptive summaries of TEAEs (graded per CTCAE v5.0) as patient counts and percentages by treatment group, with repeated events of the same type counted once per patient. Serious AEs, TEAEs leading to death, treatment modification (such as discontinuation, interruption, or reduction), study withdrawal, and DLTs were tabulated separately. Laboratory parameters, vital signs, and electrocardiograms were summarised using descriptive statistics for observed values and changes from baseline, with categorical shifts (e.g. low/normal/high or normal/abnormal) presented as counts and percentages. Clinically significant abnormalities were captured as AEs.

Efficacy analyses used descriptive statistics for ECOG performance status and Neurologic Assessment in Neuro-Oncology (NANO) scores. Tumour response was assessed using RECIST v1.1 for solid tumours or RANO criteria for brain tumours. Best overall response and disease control rate were summarised by patient counts and percentages. Progression-free survival was estimated using the Kaplan-Meier method, with medians and 95% confidence intervals reported. Patients without post-baseline tumour assessments were considered non-responders, and standard RECIST/RANO censoring rules were applied.

Supplementary Materials

Supplementary Table S1. Summary of patient data based on tumour type, efficacy time to event and best overall response

Dose and frequency	Sex (Age)	Tumour type at study entry	Locally advanced / metastatic	Efficacy time to event (weeks)	Best overall response
**Phase I**					
50 mg od	F (61)	Endometrial cancer	Metastatic	7.71	Stable disease
100 mg od	F (49)	Cervical cancer	Metastatic	6.29	Progressive disease
150 mg od	F (64)	Brain cancer (GBM)	Locally advanced	1.43	Progressive disease
	M (45)	Brain cancer (anaplastic oligodendroglioma)	Locally advanced	7.57	Progressive disease
	F (57)	Colorectal adenocarcinoma	Metastatic	7.14	Progressive disease
	F (79)	Endometrial cancer	Metastatic	6.29	Progressive disease
250 mg od	F (50)	Liver cancer	Metastatic	20.00	Stable disease
	F (54)	Brain cancer (GBM)	Locally advanced	8.00	Progressive disease
	M (66)	Pancreatic cancer	Metastatic	7.29	Progressive disease
350 mg od	M (73)	Colorectal adenocarcinoma	Metastatic	7.14	Progressive disease
	M (55)	Liver cancer	Metastatic	–	Not evaluable
	F (70)	Pancreatic cancer	Metastatic	5.57	Stable disease
175 mg b.i.d.	M (68)	Small-cell neuroendocrine carcinoma	Metastatic	3.86	Progressive disease
	F (82)	Pleural mesothelioma	Metastatic	–	Not evaluable
	M (47)	Rectosigmoid cancer	Metastatic	11.00	Progressive disease
250 mg b.i.d.	F (70)	Chordoma	Locally advanced	10.14	Progressive disease
	F (27)	LGSOC	Metastatic	17.71	Stable disease
	M (74)	Pancreatic cancer	Metastatic	20.43	Stable disease
500 mg b.i.d.	M (45)	Brain cancer (meningioma)	Locally advanced	7.14	Stable disease
	M (74)	NSCLC	Metastatic	11.71	Stable disease
**Phase IIa**					
100 mg od	M (61)	High-grade glioma (GBM)	Locally advanced	7.29	Progressive disease
150 mg od	M (62)	High-grade glioma (GBM)	Locally advanced	43.43	Stable disease
100 mg b.i.d.	M (56)	High-grade glioma (GBM)	Locally advanced	12.43	Stable disease
	M (67)	High-grade glioma (GBM)	Locally advanced	12.29	Stable disease

	F (38)	High-grade glioma (GBM)	Locally advanced	7.43	Progressive disease
	M (48)	High-grade glioma (GBM)	Locally advanced	10.00	Progressive disease
	M (65)[†]	High-grade glioma (GBM)	Locally advanced	49.29	Stable disease
150 mg b.i.d.	M (53)	High-grade glioma (GBM)	Locally advanced	7.86	Progressive disease
	M (66)	High-grade glioma (GBM)	Metastatic	3.00	Progressive disease
	F (40)[†]	High-grade glioma (unspecified)	Locally advanced	39.14	Stable disease
300 mg b.i.d.	M (46)	High-grade glioma (anaplastic astrocytoma)	Locally advanced	7.29	Stable disease
	M (48)	High-grade glioma (GBM)	Locally advanced	4.43	Progressive disease
	M (70)	High-grade glioma (GBM)	Locally advanced	7.14	Progressive disease
500 mg b.i.d.	M (61)[‡]	High-grade glioma (GBM)	Locally advanced	8.43	Stable disease
	F (44)[§]	High-grade glioma (GBM)	Locally advanced	–	Not evaluable
	M (58)	High-grade glioma (GBM)	Metastatic	–	Not evaluable
	M (70)	High-grade glioma (GBM)	Locally advanced	8.29	Progressive disease

[†]Patients continued receiving study treatment under the Special Access Scheme. [‡]Patient received re-irradiation within six months prior to the first dose of auceliciclib. [§]Patient entered the study within six months of completing radiotherapy. b.i.d., twice daily; F, female; GBM, glioblastoma; LGSOC, low-grade serous ovarian cancer; M, male; NSCLC, non-small-cell lung cancer; od, once daily; TMZ, temozolomide.

Supplementary Table S2. Summary of patient disposition

	**Phase I (*n* = 20)**	**Phase IIa (*n* = 17)**	**All (*n* = 37)**
**Number of patients treated (received at least one dose of study drug), *n* (%)**	20 (100.0)	17 (100.0)	37 (100.0)
**Number of patients discontinued treatment, *n* (%)**	20 (100.0)	17 (100.0)	37 (100.0)
**Reason for discontinued treatment, *n* (%)**			
**Adverse event**	2 (10.0)	2 (11.8)	4 (10.8)
**Death**	1 (5.0)	0 (0.0)	1 (2.7)
**Other**	1 (5.0)	2 (11.8)	3 (8.1)
**Progressive Disease**	14 (70.0)	11 (64.7)	25 (67.6)
**Withdrawal by Subject**	2 (10.0)	2 (11.8)	4 (10.8)

n, sample size.

Supplementary Table S3. Summary of treatment emergent adverse events

	**Phase I (*n* = 20) *n* (%) M†**	**Phase IIa (*n* = 17) *n* (%) M†**	**All (*n* = 37) *n* (%) M†**
Number of patients with any			
TEAEs	19 (95.0) 131	17 (100.0) 139	36 (97.3) 270
Serious TEAEs	2 (10.0) 2	3 (17.6) 4	5 (13.5) 6
TEAEs leading to death	0	0	0
TEAEs leading to study withdrawal	0	1 (5.9) 1	1 (2.7) 1
TEAEs leading to discontinuation of auceliciclib	2 (10.0) 2	2 (11.8) 3	4 (10.8) 5
TEAEs leading to discontinuation of TMZ	0	2 (11.8) 3	2 (5.4) 3
TEAEs leading to dose interruption of auceliciclib	4 (20.0) 9	1 (5.9) 2	5 (13.5) 11
TEAEs leading to dose interruption of TMZ	0	2 (11.8) 3	2 (5.4) 3
TEAEs leading to dose reduction of auceliciclib	0	1 (5.9) 2	1 (2.7) 2
TEAEs leading to dose reduction of TMZ	0	0	0
TEAEs by severity			
Mild	7 (35.0) 82	4 (23.5) 101	11 (29.7) 183
Moderate	4 (20.0) 31	7 (41.2) 25	11 (29.7) 56
Severe	8 (40.0) 18	6 (35.3) 13	14 (37.8) 31
Life-threatening	0	0	0
Death	0	0	0
TEAEs by relationship to auceliciclib			
Definitely	0	2 (11.8) 4	2 (5.4) 4
Probably	3 (15.0) 7	4 (23.5) 11	7 (18.9) 18
Possibly	11 (55.0) 31	10 (58.8) 33	21 (56.8) 64
Unlikely	3 (15.0) 28	0 (0) 32	3 (8.1) 60
Unrelated	2 (10.0) 65	1 (5.9) 59	3 (8.1) 124
TEAEs by relationship to TMZ			
Definitely	0	2 (11.8) 2	2 (5.4) 2
Probably	0	4 (23.5) 13	4 (10.8) 13
Possibly	0	10 (58.8) 32	10 (27.0) 32
Unlikely	0	0 (0) 32	0 (0) 32
Unrelated	0	1 (5.9) 60	1 (2.7) 60
Not applicable	19 (95.0) 131	0	19 (51.4) 131

†Percentages are calculated (the denominator used for the calculation) based on the number of patients of each phase. The number of patients is counted once for the highest relationship to auceliciclib for a given adverse event in patient count (*n*). Occurrences are counted each time in events (M). *n*, sample size; TEAE, treatment-emergent adverse event; TMZ, temozolomide.

Supplementary Table S4. ECOG and NANO scores of patients at baseline

ECOG performance status, *n* (%)[†]	Phase I (*n* = 18)	Phase IIa (*n* = 15)	All (*n* = 33)
0	7 (38.9)	6 (40.0)	13 (39.4)
1	10 (55.6)	8 (53.3)	18 (54.5)
2	1 (5.6)	1 (6.7)	2 (6.1)
**NANO score, *n* (%)[‡]**	**Phase I (*n* = 4)**	**Phase IIa (*n* = 14)[§]**	**All (*n* = 18)**
**Ataxia**			
0 – 2	3 (75.0)	13 (92.9)	16 (88.9)
≥ 3	0 (0.0)	0 (0.0)	0 (0.0)
Not assessed/not evaluable	1 (25.0)	1 (7.1)	2 (11.1)
**Behaviour**			
0 – 2	4 (100.0)	14 (100.0)	18 (100.0)
≥ 3	0 (0.0)	0 (0.0)	0 (0.0)
Not assessed/not evaluable	0 (0.0)	0 (0.0)	0 (0.0)
**Facial strength**			
0 – 2	4 (100.0)	14 (100.0)	18 (100.0)
≥ 3	0 (0.0)	0 (0.0)	0 (0.0)
Not assessed/not evaluable	0 (0.0)	0 (0.0)	0 (0.0)
**Gait**			
0 – 2	4 (100.0)	14 (100.0)	18 (100.0)
≥ 3	0 (0.0)	0 (0.0)	0 (0.0)
Not assessed/not evaluable	0 (0.0)	0 (0.0)	0 (0.0)
**Language**			
0 – 2	4 (100.0)	14 (100.0)	18 (100.0)
≥ 3	0 (0.0)	0 (0.0)	0 (0.0)
Not assessed/not evaluable	0 (0.0)	0 (0.0)	0 (0.0)
**Level of consciousness**			
0 – 2	4 (100.0)	14 (100.0)	18 (100.0)
≥ 3	0 (0.0)	0 (0.0)	0 (0.0)
Not assessed/not evaluable	0 (0.0)	0 (0.0)	0 (0.0)
**Sensation**			
0 – 2	4 (100.0)	14 (100.0)	18 (100.0)
≥ 3	0 (0.0)	0 (0.0)	0 (0.0)
Not assessed/not evaluable	0 (0.0)	0 (0.0)	0 (0.0)
**Strength**			
0 – 2	4 (100.0)	13 (92.9)	17 (94.4)
≥ 3	0 (0.0)	1 (7.1)	1 (5.6)
Not assessed/not evaluable	0 (0.0)	0 (0.0)	0 (0.0)
**Visual fields**			
0 – 2	3 (75.0)	11 (78.6)	14 (77.8)
≥ 3	1 (25.0)	3 (21.4)	4 (22.2)
Not assessed/not evaluable	0 (0.0)	0 (0.0)	0 (0.0)

[†]ECOG assessment is based on patients who received any amount of auceliciclib and completed a post-baseline disease response assessment. [‡]NANO assessment only for patients with brain tumour, including glioblastoma. [§]One patient did not perform assessment. ECOG, Eastern Cooperative Oncology Group; *n*, sample size; NANO, Neurologic Assessment in Neuro-Oncology.

Supplementary Table S5. ECOG performance status over time

ECOG performance status, *n* (%)[†]	Phase I	Phase IIa	All	ECOG performance status, *n* (%)[†]	Phase I	Phase IIa	All
**Cycle 1 Day 15**				**Cycle 8 Day 1**			
n	17	13	30	*n*	–	3	3
0 – 1	14 (82.4)	12 (92.3)	26 (86.7)	0 – 1	–	3 (100.0)	3 (100.0)
> 2	3 (17.6)	1 (7.7)	4 (13.3)	> 2	–	0 (0.0)	0 (0.0)
**Cycle 2 Day 1**				**Cycle 8 Day 22**			
n	16	13	29	*n*	–	3	3
0 – 1	12 (75.0)	12 (92.3)	24 (82.8)	0 – 1	–	3 (100.0)	3 (100.0)
> 2	4 (25.0)	1 (7.7)	5 (17.2)	> 2	–	0 (0.0)	0 (0.0)
**Cycle 2 Day 15**				**Cycle 9 Day 1**			
n	12	11	23	*n*	–	3	3
0 – 1	8 (66.7)	11 (100.0)	19 (82.6)	0 – 1	–	3 (100.0)	3 (100.0)
> 2	4 (33.3)	0 (0.0)	4 (17.4)	> 2	–	0 (0.0)	0 (0.0)
**Cycle 2 Day 22**				**Cycle 9 Day 22**			
n	8	9	17	*n*	–	3	3
0 – 1	7 (87.5)	7 (77.8)	14 (82.4)	0 – 1	–	3 (100.0)	3 (100.0)
> 2	1 (12.5)	2 (22.2)	3 (17.6)	> 2	–	0 (0.0)	0 (0.0)
**Cycle 3 Day 1**				**Cycle 10 Day 1**			
n	4	6	10	*n*	–	3	3
0 – 1	2 (50.0)	6 (100.0)	8 (80.0)	0 – 1	–	3 (100.0)	3 (100.0)
> 2	2 (50.0)	0 (0.0)	2 (20.0)	> 2	–	0 (0.0)	0 (0.0)
**Cycle 3 Day 22**				**Cycle 10 Day 22**			
n	2	5	7	*n*	–	3	3
0 – 1	2 (100.0)	4 (80.0)	6 (85.7)	0 – 1	–	3 (100.0)	3 (100.0)
> 2	0 (0.0)	1 (20.0)	1 (14.3)	> 2	–	0 (0.0)	0 (0.0)
**Cycle 4 Day 1**				**Cycle 11 Day 1**			
n	3	5	8	*n*	–	3	3
0 – 1	2 (66.7)	4 (80.0)	6 (75.0)	0 – 1	–	3 (100.0)	3 (100.0)
> 2	1 (33.3)	1 (20.0)	2 (25.0)	> 2	–	0 (0.0)	0 (0.0)
**Cycle 4 Day 22**				**Cycle 11 Day 22**			

n	2	4	6	*n*	–	1	1
0 – 1	1 (50.0)	4 (100.0)	5 (83.3)	0 – 1	–	1 (100.0)	1 (100.0)
> 2	1 (50.0)	0 (0.0)	1 (16.7)	> 2	–	0 (0.0)	0 (0.0)
**Cycle 5 Day 1**				**Cycle 12 Day 1**			
n	3	3	6	*n*	–	1	1
0 – 1	2 (66.7)	3 (100.0)	5 (83.3)	0 – 1	–	1 (100.0)	1 (100.0)
> 2	1 (33.3)	0 (0.0)	1 (16.7)	> 2	–	0 (0.0)	0 (0.0)
**Cycle 5 Day 22**				**Cycle 12 Day 22**			
n	1	1	2	*n*	–	1	1
0 – 1	1 (100.0)	1 (100.0)	2 (100.0)	0 – 1	–	1 (100.0)	1 (100.0)
> 2	0 (0.0)	0 (0.0)	0 (0.0)	> 2	–	0 (0.0)	0 (0.0)
**Cycle 6 Day 1**				**Cycle 13 Day 1**			
n	1	3	4	*n*	–	1	1
0 – 1	1 (100.0)	3 (100.0)	4 (100.0)	0 – 1	–	1 (100.0)	1 (100.0)
> 2	0 (0.0)	0 (0.0)	0 (0.0)	> 2	–	0 (0.0)	0 (0.0)
**Cycle 6 Day 22**				**Cycle 13 Day 22**			
n	–	3	3	*n*	–	1	1
0 – 1	–	3 (100.0)	3 (100.0)	0 – 1	–	1 (100.0)	1 (100.0)
> 2	–	0 (0.0)	0 (0.0)	> 2	–	0 (0.0)	0 (0.0)
**Cycle 7 Day 1**				**Cycle 14 Day 1**			
n	1	3	4	*n*	–	1	1
0 – 1	0 (0.0)	3 (100.0)	3 (75.0)	0 – 1	–	1 (100.0)	1 (100.0)
> 2	1 (100.0)	0 (0.0)	1 (25.0)	> 2	–	0 (0.0)	0 (0.0)
**Cycle 7 Day 22**				**Cycle 14 Day 22**			
n	–	3	3	*n*	–	1	1
0 – 1	–	3 (100.0)	3 (100.0)	0 – 1	–	1 (100.0)	1 (100.0)
> 2	–	0 (0.0)	0 (0.0)	> 2	–	0 (0.0)	0 (0.0)
				**End of Study**			
				n	8	7	15
				0 – 1	7 (87.5)	3 (42.9)	10 (66.7)
				> 2	1 (12.5)	4 (57.1)	5 (33.3)

[†]ECOG assessment is based on patients who received any amount of auceliciclib and completed a post-baseline disease response assessment. ECOG, Eastern Cooperative Oncology Group; *n*, sample size; NA, not available.

Supplementary Table S6. NANO domain scores over time

NANO score, *n* (%)†	Phase I	Phase IIa	All	NANO score, *n* (%)†	Phase I	Phase IIa	All
**Cycle 1 Day 15**				**Cycle 8 Day 1**			
**Ataxia**	3	12	15	**Ataxia**	–	3	3
0 – 2	3 (100.0)	11 (91.7)	14 (93.3)	0 – 2	–	3 (100.0)	3 (100.0)
≥ 3	0 (0.0)	0 (0.0)	0 (0.0)	≥ 3	–	0 (0.0)	0 (0.0)
Not assessed/not evaluable	0 (0.0)	1 (8.3)	1 (6.7)	Not assessed/not evaluable	–	0 (0.0)	0 (0.0)
**Behaviour**	3	12	15	**Behaviour**	–	3	3
0 – 2	3 (100.0)	12 (100.0)	15 (100.0)	0 – 2	–	3 (100.0)	3 (100.0)
≥ 3	0 (0.0)	0 (0.0)	0 (0.0)	≥ 3	–	0 (0.0)	0 (0.0)
Not assessed/not evaluable	0 (0.0)	0 (0.0)	0 (0.0)	Not assessed/not evaluable	–	0 (0.0)	0 (0.0)
**Facial strength**	3	12	15	**Facial strength**	–	3	3
0 – 2	3 (100.0)	12 (100.0)	15 (100.0)	0 – 2	–	3 (100.0)	3 (100.0)
≥ 3	0 (0.0)	0 (0.0)	0 (0.0)	≥ 3	–	0 (0.0)	0 (0.0)
Not assessed/not evaluable	0 (0.0)	0 (0.0)	0 (0.0)	Not assessed/not evaluable	–	0 (0.0)	0 (0.0)
**Gait**	3	12	15	**Gait**	–	3	3
0 – 2	3 (100.0)	12 (100.0)	15 (100.0)	0 – 2	–	3 (100.0)	3 (100.0)
≥ 3	0 (0.0)	0 (0.0)	0 (0.0)	≥ 3	–	0 (0.0)	0 (0.0)
Not assessed/not evaluable	0 (0.0)	0 (0.0)	0 (0.0)	Not assessed/not evaluable	–	0 (0.0)	0 (0.0)
**Language**	3	12	15	**Language**	–	3	3
0 – 2	3 (100.0)	12 (100.0)	15 (100.0)	0 – 2	–	3 (100.0)	3 (100.0)
≥ 3	0 (0.0)	0 (0.0)	0 (0.0)	≥ 3	–	0 (0.0)	0 (0.0)
Not assessed/not evaluable	0 (0.0)	0 (0.0)	0 (0.0)	Not assessed/not evaluable	–	0 (0.0)	0 (0.0)
**Level of consciousness**	3	12	15	**Level of consciousness**	–	3	3
0 – 2	3 (100.0)	12 (100.0)	15 (100.0)	0 – 2	–	3 (100.0)	3 (100.0)
≥ 3	0 (0.0)	0 (0.0)	0 (0.0)	≥ 3	–	0 (0.0)	0 (0.0)
Not assessed/not evaluable	0 (0.0)	0 (0.0)	0 (0.0)	Not assessed/not evaluable	–	0 (0.0)	0 (0.0)
**Sensation**	3	12	15	**Sensation**	–	3	3
0 – 2	3 (100.0)	12 (100.0)	15 (100.0)	0 – 2	–	3 (100.0)	3 (100.0)
≥ 3	0 (0.0)	0 (0.0)	0 (0.0)	≥ 3	–	0 (0.0)	0 (0.0)
Not assessed/not evaluable	0 (0.0)	0 (0.0)	0 (0.0)	Not assessed/not evaluable	–	0 (0.0)	0 (0.0)

**Strength**	3	12	15	**Strength**	–	3	3
0 – 2	3 (100.0)	11 (91.7)	14 (93.3)	0 – 2	–	3 (100.0)	3 (100.0)
≥ 3	0 (0.0)	1 (8.3)	1 (6.7)	≥ 3	–	0 (0.0)	0 (0.0)
Not assessed/not evaluable	0 (0.0)	0 (0.0)	0 (0.0)	Not assessed/not evaluable	–	0 (0.0)	0 (0.0)
**Visual fields**	3	12	15	**Visual fields**	–	3	3
0 – 2	3 (100.0)	9 (75.0)	12 (80.0)	0 – 2	–	3 (100.0)	3 (100.0)
≥ 3	0 (0.0)	3 (25.0)	3 (20.0)	≥ 3	–	0 (0.0)	0 (0.0)
Not assessed/not evaluable	0 (0.0)	0 (0.0)	0 (0.0)	Not assessed/not evaluable	–	0 (0.0)	0 (0.0)
**Cycle 2 Day 1**				**Cycle 8 Day 22**			
**Ataxia**	2	13	15	**Ataxia**	–	3	3
0 – 2	2 (100.0)	12 (92.3)	14 (93.3)	0 – 2	–	3 (100.0)	3 (100.0)
≥ 3	0 (0.0)	0 (0.0)	0 (0.0)	≥ 3	–	0 (0.0)	0 (0.0)
Not assessed/not evaluable	0 (0.0)	1 (7.7)	1 (6.7)	Not assessed/not evaluable	–	0 (0.0)	0 (0.0)
**Behaviour**	2	13	15	**Behaviour**	–	3	3
0 – 2	2 (100.0)	13 (100.0)	15 (100.0)	0 – 2	–	3 (100.0)	3 (100.0)
≥ 3	0 (0.0)	0 (0.0)	0 (0.0)	≥ 3	–	0 (0.0)	0 (0.0)
Not assessed/not evaluable	0 (0.0)	0 (0.0)	0 (0.0)	Not assessed/not evaluable	–	0 (0.0)	0 (0.0)
**Facial strength**	2	13	15	**Facial strength**	–	3	3
0 – 2	2 (100.0)	13 (100.0)	15 (100.0)	0 – 2	–	3 (100.0)	3 (100.0)
≥ 3	0 (0.0)	0 (0.0)	0 (0.0)	≥ 3	–	0 (0.0)	0 (0.0)
Not assessed/not evaluable	0 (0.0)	0 (0.0)	0 (0.0)	Not assessed/not evaluable	–	0 (0.0)	0 (0.0)
**Gait**	2	13	15	**Gait**	–	3	3
0 – 2	2 (100.0)	13 (100.0)	15 (100.0)	0 – 2	–	3 (100.0)	3 (100.0)
≥ 3	0 (0.0)	0 (0.0)	0 (0.0)	≥ 3	–	0 (0.0)	0 (0.0)
Not assessed/not evaluable	0 (0.0)	0 (0.0)	0 (0.0)	Not assessed/not evaluable	–	0 (0.0)	0 (0.0)
**Language**	2	13	15	**Language**	–	3	3
0 – 2	2 (100.0)	13 (100.0)	15 (100.0)	0 – 2	–	3 (100.0)	3 (100.0)
≥ 3	0 (0.0)	0 (0.0)	0 (0.0)	≥ 3	–	0 (0.0)	0 (0.0)
Not assessed/not evaluable	0 (0.0)	0 (0.0)	0 (0.0)	Not assessed/not evaluable	–	0 (0.0)	0 (0.0)
**Level of consciousness**	2	13	15	**Level of consciousness**	–	3	3
0 – 2	2 (100.0)	13 (100.0)	15 (100.0)	0 – 2	–	3 (100.0)	3 (100.0)
≥ 3	0 (0.0)	0 (0.0)	0 (0.0)	≥ 3	–	0 (0.0)	0 (0.0)
Not assessed/not evaluable	0 (0.0)	0 (0.0)	0 (0.0)	Not assessed/not evaluable	–	0 (0.0)	0 (0.0)
**Sensation**	2	13	15	**Sensation**	–	3	3

0 – 2	2 (100.0)	13 (100.0)	15 (100.0)	0 – 2	–	3 (100.0)	3 (100.0)
≥ 3	0 (0.0)	0 (0.0)	0 (0.0)	≥ 3	–	0 (0.0)	0 (0.0)
Not assessed/not evaluable	0 (0.0)	0 (0.0)	0 (0.0)	Not assessed/not evaluable	–	0 (0.0)	0 (0.0)
**Strength**	2	13	15	**Strength**	–	3	3
0 – 2	2 (100.0)	12 (92.3)	14 (93.3)	0 – 2	–	3 (100.0)	3 (100.0)
≥ 3	0 (0.0)	1 (7.7)	1 (6.7)	≥ 3	–	0 (0.0)	0 (0.0)
Not assessed/not evaluable	0 (0.0)	0 (0.0)	0 (0.0)	Not assessed/not evaluable	–	0 (0.0)	0 (0.0)
**Visual fields**	2	13	15	**Visual fields**	–	3	3
0 – 2	2 (100.0)	10 (76.9)	12 (80.0)	0 – 2	–	3 (100.0)	3 (100.0)
≥ 3	0 (0.0)	3 (23.1)	3 (20.0)	≥ 3	–	0 (0.0)	0 (0.0)
Not assessed/not evaluable	0 (0.0)	0 (0.0)	0 (0.0)	Not assessed/not evaluable	–	0 (0.0)	0 (0.0)
**Cycle 2 Day 15**				**Cycle 9 Day 1**			
**Ataxia**	2	10	12	**Ataxia**	–	3	3
0 – 2	2 (100.0)	9 (90.0)	11 (91.7)	0 – 2	–	3 (100.0)	3 (100.0)
≥ 3	0 (0.0)	0 (0.0)	0 (0.0)	≥ 3	–	0 (0.0)	0 (0.0)
Not assessed/not evaluable	0 (0.0)	1 (10.0)	1 (8.3)	Not assessed/not evaluable	–	0 (0.0)	0 (0.0)
**Behaviour**	2	10	12	**Behaviour**	–	3	3
0 – 2	2 (100.0)	10 (100.0)	12 (100.0)	0 – 2	–	3 (100.0)	3 (100.0)
≥ 3	0 (0.0)	0 (0.0)	0 (0.0)	≥ 3	–	0 (0.0)	0 (0.0)
Not assessed/not evaluable	0 (0.0)	0 (0.0)	0 (0.0)	Not assessed/not evaluable	–	0 (0.0)	0 (0.0)
**Facial strength**	2	10	12	**Facial strength**	–	3	3
0 – 2	2 (100.0)	10 (100.0)	12 (100.0)	0 – 2	–	3 (100.0)	3 (100.0)
≥ 3	0 (0.0)	0 (0.0)	0 (0.0)	≥ 3	–	0 (0.0)	0 (0.0)
Not assessed/not evaluable	0 (0.0)	0 (0.0)	0 (0.0)	Not assessed/not evaluable	–	0 (0.0)	0 (0.0)
**Gait**	2	10	12	**Gait**	–	3	3
0 – 2	2 (100.0)	10 (100.0)	12 (100.0)	0 – 2	–	3 (100.0)	3 (100.0)
≥ 3	0 (0.0)	0 (0.0)	0 (0.0)	≥ 3	–	0 (0.0)	0 (0.0)
Not assessed/not evaluable	0 (0.0)	0 (0.0)	0 (0.0)	Not assessed/not evaluable	–	0 (0.0)	0 (0.0)
**Language**	2	10	12	**Language**	–	3	3
0 – 2	2 (100.0)	10 (100.0)	12 (100.0)	0 – 2	–	3 (100.0)	3 (100.0)
≥ 3	0 (0.0)	0 (0.0)	0 (0.0)	≥ 3	–	0 (0.0)	0 (0.0)
Not assessed/not evaluable	0 (0.0)	0 (0.0)	0 (0.0)	Not assessed/not evaluable	–	0 (0.0)	0 (0.0)
**Level of consciousness**	2	10	12	**Level of consciousness**	–	3	3
0 – 2	2 (100.0)	10 (100.0)	12 (100.0)	0 – 2	–	3 (100.0)	3 (100.0)

≥ 3	0 (0.0)	0 (0.0)	0 (0.0)	≥ 3	–	0 (0.0)	0 (0.0)
Not assessed/not evaluable	0 (0.0)	0 (0.0)	0 (0.0)	Not assessed/not evaluable	–	0 (0.0)	0 (0.0)
**Sensation**	2	10	12	**Sensation**	–	3	3
0 – 2	2 (100.0)	10 (100.0)	12 (100.0)	0 – 2	–	3 (100.0)	3 (100.0)
≥ 3	0 (0.0)	0 (0.0)	0 (0.0)	≥ 3	–	0 (0.0)	0 (0.0)
Not assessed/not evaluable	0 (0.0)	0 (0.0)	0 (0.0)	Not assessed/not evaluable	–	0 (0.0)	0 (0.0)
**Strength**	2	10	12	**Strength**	–	3	3
0 – 2	2 (100.0)	9 (90.0)	11 (91.7)	0 – 2	–	3 (100.0)	3 (100.0)
≥ 3	0 (0.0)	1 (10.0)	1 (8.3)	≥ 3	–	0 (0.0)	0 (0.0)
Not assessed/not evaluable	0 (0.0)	0 (0.0)	0 (0.0)	Not assessed/not evaluable	–	0 (0.0)	0 (0.0)
**Visual fields**	2	10	12	**Visual fields**	–	3	3
0 – 2	2 (100.0)	9 (90.0)	11 (91.7)	0 – 2	–	3 (100.0)	3 (100.0)
≥ 3	0 (0.0)	1 (10.0)	1 (8.3)	≥ 3	–	0 (0.0)	0 (0.0)
Not assessed/not evaluable	0 (0.0)	0 (0.0)	0 (0.0)	Not assessed/not evaluable	–	0 (0.0)	0 (0.0)
**Cycle 2 Day 22**				**Cycle 9 Day 22**			
**Ataxia**	–	8	8	**Ataxia**	–	2	2
0 – 2	–	7 (87.5)	7 (87.5)	0 – 2	–	2 (100.0)	2 (100.0)
≥ 3	–	0 (0.0)	0 (0.0)	≥ 3	–	0 (0.0)	0 (0.0)
Not assessed/not evaluable	–	1 (12.5)	1 (12.5)	Not assessed/not evaluable	–	0 (0.0)	0 (0.0)
**Behaviour**	–	8	8	**Behaviour**	–	2	2
0 – 2	–	8 (100.0)	8 (100.0)	0 – 2	–	2 (100.0)	2 (100.0)
≥ 3	–	0 (0.0)	0 (0.0)	≥ 3	–	0 (0.0)	0 (0.0)
Not assessed/not evaluable	–	0 (0.0)	0 (0.0)	Not assessed/not evaluable	–	0 (0.0)	0 (0.0)
**Facial strength**	–	8	8	**Facial strength**	–	2	2
0 – 2	–	8 (100.0)	8 (100.0)	0 – 2	–	2 (100.0)	2 (100.0)
≥ 3	–	0 (0.0)	0 (0.0)	≥ 3	–	0 (0.0)	0 (0.0)
Not assessed/not evaluable	–	0 (0.0)	0 (0.0)	Not assessed/not evaluable	–	0 (0.0)	0 (0.0)
**Gait**	–	8	8	**Gait**	–	2	2
0 – 2	–	8 (100.0)	8 (100.0)	0 – 2	–	2 (100.0)	2 (100.0)
≥ 3	–	0 (0.0)	0 (0.0)	≥ 3	–	0 (0.0)	0 (0.0)
Not assessed/not evaluable	–	0 (0.0)	0 (0.0)	Not assessed/not evaluable	–	0 (0.0)	0 (0.0)
**Language**	–	8	8	**Language**	–	2	2
0 – 2	–	8 (100.0)	8 (100.0)	0 – 2	–	2 (100.0)	2 (100.0)
≥ 3	–	0 (0.0)	0 (0.0)	≥ 3	–	0 (0.0)	0 (0.0)

Not assessed/not evaluable	–	0 (0.0)	0 (0.0)	Not assessed/not evaluable	–	0 (0.0)	0 (0.0)
**Level of consciousness**	–	8	8	**Level of consciousness**	–	2	2
0 – 2	–	8 (100.0)	8 (100.0)	0 – 2	–	2 (100.0)	2 (100.0)
≥ 3	–	0 (0.0)	0 (0.0)	≥ 3	–	0 (0.0)	0 (0.0)
Not assessed/not evaluable	–	0 (0.0)	0 (0.0)	Not assessed/not evaluable	–	0 (0.0)	0 (0.0)
**Sensation**	–	8	8	**Sensation**	–	2	2
0 – 2	–	8 (100.0)	8 (100.0)	0 – 2	–	2 (100.0)	2 (100.0)
≥ 3	–	0 (0.0)	0 (0.0)	≥ 3	–	0 (0.0)	0 (0.0)
Not assessed/not evaluable	–	0 (0.0)	0 (0.0)	Not assessed/not evaluable	–	0 (0.0)	0 (0.0)
**Strength**	–	8	8	**Strength**	–	2	2
0 – 2	–	7 (87.5)	7 (87.5)	0 – 2	–	2 (100.0)	2 (100.0)
≥ 3	–	1 (12.5)	1 (12.5)	≥ 3	–	0 (0.0)	0 (0.0)
Not assessed/not evaluable	–	0 (0.0)	0 (0.0)	Not assessed/not evaluable	–	0 (0.0)	0 (0.0)
**Visual fields**	–	8	8	**Visual fields**	–	2	2
0 – 2	–	8 (100.0)	8 (100.0)	0 – 2	–	2 (100.0)	2 (100.0)
≥ 3	–	0 (0.0)	0 (0.0)	≥ 3	–	0 (0.0)	0 (0.0)
Not assessed/not evaluable	–	0 (0.0)	0 (0.0)	Not assessed/not evaluable	–	0 (0.0)	0 (0.0)
**Cycle 3 Day 1**				**Cycle 10 Day 1**			
**Ataxia**	–	5	5	**Ataxia**	–	3	3
0 – 2	–	4 (80.0)	4 (80.0)	0 – 2	–	3 (100.0)	3 (100.0)
≥ 3	–	0 (0.0)	0 (0.0)	≥ 3	–	0 (0.0)	0 (0.0)
Not assessed/not evaluable	–	1 (20.0)	1 (20.0)	Not assessed/not evaluable	–	0 (0.0)	0 (0.0)
**Behaviour**	–	5	5	**Behaviour**	–	3	3
0 – 2	–	5 (100.0)	5 (100.0)	0 – 2	–	3 (100.0)	3 (100.0)
≥ 3	–	0 (0.0)	0 (0.0)	≥ 3	–	0 (0.0)	0 (0.0)
Not assessed/not evaluable	–	0 (0.0)	0 (0.0)	Not assessed/not evaluable	–	0 (0.0)	0 (0.0)
**Facial strength**	–	5	5	**Facial strength**	–	3	3
0 – 2	–	5 (100.0)	5 (100.0)	0 – 2	–	3 (100.0)	3 (100.0)
≥ 3	–	0 (0.0)	0 (0.0)	≥ 3	–	0 (0.0)	0 (0.0)
Not assessed/not evaluable	–	0 (0.0)	0 (0.0)	Not assessed/not evaluable	–	0 (0.0)	0 (0.0)
**Gait**	–	5	5	**Gait**	–	3	3
0 – 2	–	4 (80.0)	4 (80.0)	0 – 2	–	3 (100.0)	3 (100.0)
≥ 3	–	1 (20.0)	1 (20.0)	≥ 3	–	0 (0.0)	0 (0.0)
Not assessed/not evaluable	–	0 (0.0)	0 (0.0)	Not assessed/not evaluable	–	0 (0.0)	0 (0.0)

**Language**	–	5	5	**Language**	–	3	3
0 – 2	–	5 (100.0)	5 (100.0)	0 – 2	–	3 (100.0)	3 (100.0)
≥ 3	–	0 (0.0)	0 (0.0)	≥ 3	–	0 (0.0)	0 (0.0)
Not assessed/not evaluable	–	0 (0.0)	0 (0.0)	Not assessed/not evaluable	–	0 (0.0)	0 (0.0)
**Level of consciousness**	–	5	5	**Level of consciousness**	–	3	3
0 – 2	–	5 (100.0)	5 (100.0)	0 – 2	–	3 (100.0)	3 (100.0)
≥ 3	–	0 (0.0)	0 (0.0)	≥ 3	–	0 (0.0)	0 (0.0)
Not assessed/not evaluable	–	0 (0.0)	0 (0.0)	Not assessed/not evaluable	–	0 (0.0)	0 (0.0)
**Sensation**	–	5	5	**Sensation**	–	3	3
0 – 2	–	5 (100.0)	5 (100.0)	0 – 2	–	3 (100.0)	3 (100.0)
≥ 3	–	0 (0.0)	0 (0.0)	≥ 3	–	0 (0.0)	0 (0.0)
Not assessed/not evaluable	–	0 (0.0)	0 (0.0)	Not assessed/not evaluable	–	0 (0.0)	0 (0.0)
**Strength**	–	5	5	**Strength**	–	3	3
0 – 2	–	5 (100.0)	5 (100.0)	0 – 2	–	3 (100.0)	3 (100.0)
≥ 3	–	0 (0.0)	0 (0.0)	≥ 3	–	0 (0.0)	0 (0.0)
Not assessed/not evaluable	–	0 (0.0)	0 (0.0)	Not assessed/not evaluable	–	0 (0.0)	0 (0.0)
**Visual fields**	–	5	5	**Visual fields**	–	3	3
0 – 2	–	4 (80.0)	5 (100.0)	0 – 2	–	3 (100.0)	3 (100.0)
≥ 3	–	1 (20.0)	0 (0.0)	≥ 3	–	0 (0.0)	0 (0.0)
Not assessed/not evaluable	–	0 (0.0)	0 (0.0)	Not assessed/not evaluable	–	0 (0.0)	0 (0.0)
**Cycle 3 Day 22**				**Cycle 10 Day 22**			
**Ataxia**	–	5	5	**Ataxia**	–	3	3
0 – 2	–	4 (80.0)	4 (80.0)	0 – 2	–	3 (100.0)	3 (100.0)
≥ 3	–	0 (0.0)	0 (0.0)	≥ 3	–	0 (0.0)	0 (0.0)
Not assessed/not evaluable	–	1 (20.0)	1 (20.0)	Not assessed/not evaluable	–	0 (0.0)	0 (0.0)
**Behaviour**	–	5	5	**Behaviour**	–	3	3
0 – 2	–	5 (100.0)	5 (100.0)	0 – 2	–	3 (100.0)	3 (100.0)
≥ 3	–	0 (0.0)	0 (0.0)	≥ 3	–	0 (0.0)	0 (0.0)
Not assessed/not evaluable	–	0 (0.0)	0 (0.0)	Not assessed/not evaluable	–	0 (0.0)	0 (0.0)
**Facial strength**	–	5	5	**Facial strength**	–	3	3
0 – 2	–	5 (100.0)	5 (100.0)	0 – 2	–	3 (100.0)	3 (100.0)
≥ 3	–	0 (0.0)	0 (0.0)	≥ 3	–	0 (0.0)	0 (0.0)
Not assessed/not evaluable	–	0 (0.0)	0 (0.0)	Not assessed/not evaluable	–	0 (0.0)	0 (0.0)
**Gait**	–	5	5	**Gait**	–	3	3

0 – 2	–	5 (100.0)	5 (100.0)	0 – 2	–	3 (100.0)	3 (100.0)
≥ 3	–	0 (0.0)	0 (0.0)	≥ 3	–	0 (0.0)	0 (0.0)
Not assessed/not evaluable	–	0 (0.0)	0 (0.0)	Not assessed/not evaluable	–	0 (0.0)	0 (0.0)
**Language**	–	5	5	**Language**	–	3	3
0 – 2	–	5 (100.0)	5 (100.0)	0 – 2	–	3 (100.0)	3 (100.0)
≥ 3	–	0 (0.0)	0 (0.0)	≥ 3	–	0 (0.0)	0 (0.0)
Not assessed/not evaluable	–	0 (0.0)	0 (0.0)	Not assessed/not evaluable	–	0 (0.0)	0 (0.0)
**Level of consciousness**	–	5	5	**Level of consciousness**	–	3	3
0 – 2	–	5 (100.0)	5 (100.0)	0 – 2	–	3 (100.0)	3 (100.0)
≥ 3	–	0 (0.0)	0 (0.0)	≥ 3	–	0 (0.0)	0 (0.0)
Not assessed/not evaluable	–	0 (0.0)	0 (0.0)	Not assessed/not evaluable	–	0 (0.0)	0 (0.0)
**Sensation**	–	5	5	**Sensation**	–	3	3
0 – 2	–	5 (100.0)	5 (100.0)	0 – 2	–	3 (100.0)	3 (100.0)
≥ 3	–	0 (0.0)	0 (0.0)	≥ 3	–	0 (0.0)	0 (0.0)
Not assessed/not evaluable	–	0 (0.0)	0 (0.0)	Not assessed/not evaluable	–	0 (0.0)	0 (0.0)
**Strength**	–	5	5	**Strength**	–	3	3
0 – 2	–	4 (80.0)	4 (80.0)	0 – 2	–	3 (100.0)	3 (100.0)
≥ 3	–	1 (20.0)	1 (20.0)	≥ 3	–	0 (0.0)	0 (0.0)
Not assessed/not evaluable	–	0 (0.0)	0 (0.0)	Not assessed/not evaluable	–	0 (0.0)	0 (0.0)
**Visual fields**	–	5	5	**Visual fields**	–	3	3
0 – 2	–	5 (100.0)	5 (100.0)	0 – 2	–	3 (100.0)	3 (100.0)
≥ 3	–	0 (0.0)	0 (0.0)	≥ 3	–	0 (0.0)	0 (0.0)
Not assessed/not evaluable	–	0 (0.0)	0 (0.0)	Not assessed/not evaluable	–	0 (0.0)	0 (0.0)
**Cycle 4 Day 1**				**Cycle 11 Day 1**			
**Ataxia**	–	5	5	**Ataxia**	–	3	3
0 – 2	–	5 (100.0)	5 (100.0)	0 – 2	–	3 (100.0)	3 (100.0)
≥ 3	–	0 (0.0)	0 (0.0)	≥ 3	–	0 (0.0)	0 (0.0)
Not assessed/not evaluable	–	0 (0.0)	0 (0.0)	Not assessed/not evaluable	–	0 (0.0)	0 (0.0)
**Behaviour**	–	5	5	**Behaviour**	–	3	3
0 – 2	–	5 (100.0)	5 (100.0)	0 – 2	–	3 (100.0)	3 (100.0)
≥ 3	–	0 (0.0)	0 (0.0)	≥ 3	–	0 (0.0)	0 (0.0)
Not assessed/not evaluable	–	0 (0.0)	0 (0.0)	Not assessed/not evaluable	–	0 (0.0)	0 (0.0)
**Facial strength**	–	5	5	**Facial strength**	–	3	3
0 – 2	–	5 (100.0)	5 (100.0)	0 – 2	–	3 (100.0)	3 (100.0)

≥ 3	–	0 (0.0)	0 (0.0)	≥ 3	–	0 (0.0)	0 (0.0)
Not assessed/not evaluable	–	0 (0.0)	0 (0.0)	Not assessed/not evaluable	–	0 (0.0)	0 (0.0)
**Gait**	–	5	5	**Gait**	–	3	3
0 – 2	–	4 (80.0)	4 (80.0)	0 – 2	–	3 (100.0)	3 (100.0)
≥ 3	–	1 (20.0)	1 (20.0)	≥ 3	–	0 (0.0)	0 (0.0)
Not assessed/not evaluable	–	0 (0.0)	0 (0.0)	Not assessed/not evaluable	–	0 (0.0)	0 (0.0)
**Language**	–	5	5	**Language**	–	3	3
0 – 2	–	5 (100.0)	5 (100.0)	0 – 2	–	3 (100.0)	3 (100.0)
≥ 3	–	0 (0.0)	0 (0.0)	≥ 3	–	0 (0.0)	0 (0.0)
Not assessed/not evaluable	–	0 (0.0)	0 (0.0)	Not assessed/not evaluable	–	0 (0.0)	0 (0.0)
**Level of consciousness**	–	5	5	**Level of consciousness**	–	3	3
0 – 2	–	5 (100.0)	5 (100.0)	0 – 2	–	3 (100.0)	3 (100.0)
≥ 3	–	0 (0.0)	0 (0.0)	≥ 3	–	0 (0.0)	0 (0.0)
Not assessed/not evaluable	–	0 (0.0)	0 (0.0)	Not assessed/not evaluable	–	0 (0.0)	0 (0.0)
**Sensation**	–	5	5	**Sensation**	–	3	3
0 – 2	–	5 (100.0)	5 (100.0)	0 – 2	–	3 (100.0)	3 (100.0)
≥ 3	–	0 (0.0)	0 (0.0)	≥ 3	–	0 (0.0)	0 (0.0)
Not assessed/not evaluable	–	0 (0.0)	0 (0.0)	Not assessed/not evaluable	–	0 (0.0)	0 (0.0)
**Strength**	–	5	5	**Strength**	–	3	3
0 – 2	–	5 (100.0)	5 (100.0)	0 – 2	–	3 (100.0)	3 (100.0)
≥ 3	–	0 (0.0)	0 (0.0)	≥ 3	–	0 (0.0)	0 (0.0)
Not assessed/not evaluable	–	0 (0.0)	0 (0.0)	Not assessed/not evaluable	–	0 (0.0)	0 (0.0)
**Visual fields**	–	5	5	**Visual fields**	–	3	3
0 – 2	–	4 (80.0)	4 (80.0)	0 – 2	–	3 (100.0)	3 (100.0)
≥ 3	–	1 (20.0)	1 (20.0)	≥ 3	–	0 (0.0)	0 (0.0)
Not assessed/not evaluable	–	0 (0.0)	0 (0.0)	Not assessed/not evaluable	–	0 (0.0)	0 (0.0)
**Cycle 4 Day 22**				**Cycle 11 Day 22**			
**Ataxia**	–	4	4	**Ataxia**	–	1	1
0 – 2	–	4 (100.0)	4 (100.0)	0 – 2	–	1 (100.0)	1 (100.0)
≥ 3	–	0 (0.0)	0 (0.0)	≥ 3	–	0 (0.0)	0 (0.0)
Not assessed/not evaluable	–	0 (0.0)	0 (0.0)	Not assessed/not evaluable	–	0 (0.0)	0 (0.0)
**Behaviour**	–	4	4	**Behaviour**	–	1	1
0 – 2	–	4 (100.0)	4 (100.0)	0 – 2	–	1 (100.0)	1 (100.0)
≥ 3	–	0 (0.0)	0 (0.0)	≥ 3	–	0 (0.0)	0 (0.0)

Not assessed/not evaluable	–	0 (0.0)	0 (0.0)	Not assessed/not evaluable	–	0 (0.0)	0 (0.0)
**Facial strength**	–	4	4	**Facial strength**	–	1	1
0 – 2	–	4 (100.0)	4 (100.0)	0 – 2	–	1 (100.0)	1 (100.0)
≥ 3	–	0 (0.0)	0 (0.0)	≥ 3	–	0 (0.0)	0 (0.0)
Not assessed/not evaluable	–	0 (0.0)	0 (0.0)	Not assessed/not evaluable	–	0 (0.0)	0 (0.0)
**Gait**	–	4	4	**Gait**	–	1	1
0 – 2	–	4 (100.0)	4 (100.0)	0 – 2	–	1 (100.0)	1 (100.0)
≥ 3	–	0 (0.0)	0 (0.0)	≥ 3	–	0 (0.0)	0 (0.0)
Not assessed/not evaluable	–	0 (0.0)	0 (0.0)	Not assessed/not evaluable	–	0 (0.0)	0 (0.0)
**Language**	–	4	4	**Language**	–	1	1
0 – 2	–	4 (100.0)	4 (100.0)	0 – 2	–	1 (100.0)	1 (100.0)
≥ 3	–	0 (0.0)	0 (0.0)	≥ 3	–	0 (0.0)	0 (0.0)
Not assessed/not evaluable	–	0 (0.0)	0 (0.0)	Not assessed/not evaluable	–	0 (0.0)	0 (0.0)
**Level of consciousness**	–	4	4	**Level of consciousness**	–	1	1
0 – 2	–	4 (100.0)	4 (100.0)	0 – 2	–	1 (100.0)	1 (100.0)
≥ 3	–	0 (0.0)	0 (0.0)	≥ 3	–	0 (0.0)	0 (0.0)
Not assessed/not evaluable	–	0 (0.0)	0 (0.0)	Not assessed/not evaluable	–	0 (0.0)	0 (0.0)
**Sensation**	–	4	4	**Sensation**	–	1	1
0 – 2	–	4 (100.0)	4 (100.0)	0 – 2	–	1 (100.0)	1 (100.0)
≥ 3	–	0 (0.0)	0 (0.0)	≥ 3	–	0 (0.0)	0 (0.0)
Not assessed/not evaluable	–	0 (0.0)	0 (0.0)	Not assessed/not evaluable	–	0 (0.0)	0 (0.0)
**Strength**	–	4	4	**Strength**	–	1	1
0 – 2	–	4 (100.0)	4 (100.0)	0 – 2	–	1 (100.0)	1 (100.0)
≥ 3	–	0 (0.0)	0 (0.0)	≥ 3	–	0 (0.0)	0 (0.0)
Not assessed/not evaluable	–	0 (0.0)	0 (0.0)	Not assessed/not evaluable	–	0 (0.0)	0 (0.0)
**Visual fields**	–	4	4	**Visual fields**	–	1	1
0 – 2	–	4 (100.0)	4 (100.0)	0 – 2	–	1 (100.0)	1 (100.0)
≥ 3	–	0 (0.0)	0 (0.0)	≥ 3	–	0 (0.0)	0 (0.0)
Not assessed/not evaluable	–	0 (0.0)	0 (0.0)	Not assessed/not evaluable	–	0 (0.0)	0 (0.0)
**Cycle 5 Day 1**				**Cycle 12 Day 1**			
**Ataxia**	–	3	3	**Ataxia**	–	1	1
0 – 2	–	3 (100.0)	3 (100.0)	0 – 2	–	1 (100.0)	1 (100.0)
≥ 3	–	0 (0.0)	0 (0.0)	≥ 3	–	0 (0.0)	0 (0.0)
Not assessed/not evaluable	–	0 (0.0)	0 (0.0)	Not assessed/not evaluable	–	0 (0.0)	0 (0.0)

**Behaviour**	–	3	3	**Behaviour**	–	1	1
0 – 2	–	3 (100.0)	3 (100.0)	0 – 2	–	1 (100.0)	1 (100.0)
≥ 3	–	0 (0.0)	0 (0.0)	≥ 3	–	0 (0.0)	0 (0.0)
Not assessed/not evaluable	–	0 (0.0)	0 (0.0)	Not assessed/not evaluable	–	0 (0.0)	0 (0.0)
**Facial strength**	–	3	3	**Facial strength**	–	1	1
0 – 2	–	3 (100.0)	3 (100.0)	0 – 2	–	1 (100.0)	1 (100.0)
≥ 3	–	0 (0.0)	0 (0.0)	≥ 3	–	0 (0.0)	0 (0.0)
Not assessed/not evaluable	–	0 (0.0)	0 (0.0)	Not assessed/not evaluable	–	0 (0.0)	0 (0.0)
**Gait**	–	3	3	**Gait**	–	1	1
0 – 2	–	3 (100.0)	3 (100.0)	0 – 2	–	1 (100.0)	1 (100.0)
≥ 3	–	0 (0.0)	0 (0.0)	≥ 3	–	0 (0.0)	0 (0.0)
Not assessed/not evaluable	–	0 (0.0)	0 (0.0)	Not assessed/not evaluable	–	0 (0.0)	0 (0.0)
**Language**	–	3	3	**Language**	–	1	1
0 – 2	–	3 (100.0)	3 (100.0)	0 – 2	–	1 (100.0)	1 (100.0)
≥ 3	–	0 (0.0)	0 (0.0)	≥ 3	–	0 (0.0)	0 (0.0)
Not assessed/not evaluable	–	0 (0.0)	0 (0.0)	Not assessed/not evaluable	–	0 (0.0)	0 (0.0)
**Level of consciousness**	–	3	3	**Level of consciousness**	–	1	1
0 – 2	–	3 (100.0)	3 (100.0)	0 – 2	–	1 (100.0)	1 (100.0)
≥ 3	–	0 (0.0)	0 (0.0)	≥ 3	–	0 (0.0)	0 (0.0)
Not assessed/not evaluable	–	0 (0.0)	0 (0.0)	Not assessed/not evaluable	–	0 (0.0)	0 (0.0)
**Sensation**	–	3	3	**Sensation**	–	1	1
0 – 2	–	3 (100.0)	3 (100.0)	0 – 2	–	1 (100.0)	1 (100.0)
≥ 3	–	0 (0.0)	0 (0.0)	≥ 3	–	0 (0.0)	0 (0.0)
Not assessed/not evaluable	–	0 (0.0)	0 (0.0)	Not assessed/not evaluable	–	0 (0.0)	0 (0.0)
**Strength**	–	3	3	**Strength**	–	1	1
0 – 2	–	3 (100.0)	3 (100.0)	0 – 2	–	1 (100.0)	1 (100.0)
≥ 3	–	0 (0.0)	0 (0.0)	≥ 3	–	0 (0.0)	0 (0.0)
Not assessed/not evaluable	–	0 (0.0)	0 (0.0)	Not assessed/not evaluable	–	0 (0.0)	0 (0.0)
**Visual fields**	–	3	3	**Visual fields**	–	1	1
0 – 2	–	3 (100.0)	3 (100.0)	0 – 2	–	1 (100.0)	1 (100.0)
≥ 3	–	0 (0.0)	0 (0.0)	≥ 3	–	0 (0.0)	0 (0.0)
Not assessed/not evaluable	–	0 (0.0)	0 (0.0)	Not assessed/not evaluable	–	0 (0.0)	0 (0.0)
**Cycle 5 Day 22**				**Cycle 12 Day 22**			
**Ataxia**	–	2	2	**Ataxia**	–	1	1

0 – 2	–	2 (100.0)	2 (100.0)	0 – 2	–	1 (100.0)	1 (100.0)
≥ 3	–	0 (0.0)	0 (0.0)	≥ 3	–	0 (0.0)	0 (0.0)
Not assessed/not evaluable	–	0 (0.0)	0 (0.0)	Not assessed/not evaluable	–	0 (0.0)	0 (0.0)
**Behaviour**	–	2	2	**Behaviour**	–	1	1
0 – 2	–	2 (100.0)	2 (100.0)	0 – 2	–	1 (100.0)	1 (100.0)
≥ 3	–	0 (0.0)	0 (0.0)	≥ 3	–	0 (0.0)	0 (0.0)
Not assessed/not evaluable	–	0 (0.0)	0 (0.0)	Not assessed/not evaluable	–	0 (0.0)	0 (0.0)
**Facial strength**	–	2	2	**Facial strength**	–	1	1
0 – 2	–	2 (100.0)	2 (100.0)	0 – 2	–	1 (100.0)	1 (100.0)
≥ 3	–	0 (0.0)	0 (0.0)	≥ 3	–	0 (0.0)	0 (0.0)
Not assessed/not evaluable	–	0 (0.0)	0 (0.0)	Not assessed/not evaluable	–	0 (0.0)	0 (0.0)
**Gait**	–	2	2	**Gait**	–	1	1
0 – 2	–	2 (100.0)	2 (100.0)	0 – 2	–	1 (100.0)	1 (100.0)
≥ 3	–	0 (0.0)	0 (0.0)	≥ 3	–	0 (0.0)	0 (0.0)
Not assessed/not evaluable	–	0 (0.0)	0 (0.0)	Not assessed/not evaluable	–	0 (0.0)	0 (0.0)
**Language**	–	2	2	**Language**	–	1	1
0 – 2	–	2 (100.0)	2 (100.0)	0 – 2	–	1 (100.0)	1 (100.0)
≥ 3	–	0 (0.0)	0 (0.0)	≥ 3	–	0 (0.0)	0 (0.0)
Not assessed/not evaluable	–	0 (0.0)	0 (0.0)	Not assessed/not evaluable	–	0 (0.0)	0 (0.0)
**Level of consciousness**	–	2	2	**Level of consciousness**	–	1	1
0 – 2	–	2 (100.0)	2 (100.0)	0 – 2	–	1 (100.0)	1 (100.0)
≥ 3	–	0 (0.0)	0 (0.0)	≥ 3	–	0 (0.0)	0 (0.0)
Not assessed/not evaluable	–	0 (0.0)	0 (0.0)	Not assessed/not evaluable	–	0 (0.0)	0 (0.0)
**Sensation**	–	2	2	**Sensation**	–	1	1
0 – 2	–	2 (100.0)	2 (100.0)	0 – 2	–	1 (100.0)	1 (100.0)
≥ 3	–	0 (0.0)	0 (0.0)	≥ 3	–	0 (0.0)	0 (0.0)
Not assessed/not evaluable	–	0 (0.0)	0 (0.0)	Not assessed/not evaluable	–	0 (0.0)	0 (0.0)
**Strength**	–	2	2	**Strength**	–	1	1
0 – 2	–	2 (100.0)	2 (100.0)	0 – 2	–	1 (100.0)	1 (100.0)
≥ 3	–	0 (0.0)	0 (0.0)	≥ 3	–	0 (0.0)	0 (0.0)
Not assessed/not evaluable	–	0 (0.0)	0 (0.0)	Not assessed/not evaluable	–	0 (0.0)	0 (0.0)
**Visual fields**	–	2	2	**Visual fields**	–	1	1
0 – 2	–	2 (100.0)	2 (100.0)	0 – 2	–	1 (100.0)	1 (100.0)
≥ 3	–	0 (0.0)	0 (0.0)	≥ 3	–	0 (0.0)	0 (0.0)

Not assessed/not evaluable	–	0 (0.0)	0 (0.0)	Not assessed/not evaluable	–	0 (0.0)	0 (0.0)
**Cycle 6 Day 1**				**Cycle 13 Day 1**			
**Ataxia**	–	3	3	**Ataxia**	–	1	1
0 – 2	–	3 (100.0)	3 (100.0)	0 – 2	–	1 (100.0)	1 (100.0)
≥ 3	–	0 (0.0)	0 (0.0)	≥ 3	–	0 (0.0)	0 (0.0)
Not assessed/not evaluable	–	0 (0.0)	0 (0.0)	Not assessed/not evaluable	–	0 (0.0)	0 (0.0)
**Behaviour**	–	3	3	**Behaviour**	–	1	1
0 – 2	–	3 (100.0)	3 (100.0)	0 – 2	–	1 (100.0)	1 (100.0)
≥ 3	–	0 (0.0)	0 (0.0)	≥ 3	–	0 (0.0)	0 (0.0)
Not assessed/not evaluable	–	0 (0.0)	0 (0.0)	Not assessed/not evaluable	–	0 (0.0)	0 (0.0)
**Facial strength**	–	3	3	**Facial strength**	–	1	1
0 – 2	–	3 (100.0)	3 (100.0)	0 – 2	–	1 (100.0)	1 (100.0)
≥ 3	–	0 (0.0)	0 (0.0)	≥ 3	–	0 (0.0)	0 (0.0)
Not assessed/not evaluable	–	0 (0.0)	0 (0.0)	Not assessed/not evaluable	–	0 (0.0)	0 (0.0)
**Gait**	–	3	3	**Gait**	–	1	1
0 – 2	–	3 (100.0)	3 (100.0)	0 – 2	–	1 (100.0)	1 (100.0)
≥ 3	–	0 (0.0)	0 (0.0)	≥ 3	–	0 (0.0)	0 (0.0)
Not assessed/not evaluable	–	0 (0.0)	0 (0.0)	Not assessed/not evaluable	–	0 (0.0)	0 (0.0)
**Language**	–	3	3	**Language**	–	1	1
0 – 2	–	3 (100.0)	3 (100.0)	0 – 2	–	1 (100.0)	1 (100.0)
≥ 3	–	0 (0.0)	0 (0.0)	≥ 3	–	0 (0.0)	0 (0.0)
Not assessed/not evaluable	–	0 (0.0)	0 (0.0)	Not assessed/not evaluable	–	0 (0.0)	0 (0.0)
**Level of consciousness**	–	3	3	**Level of consciousness**	–	1	1
0 – 2	–	3 (100.0)	3 (100.0)	0 – 2	–	1 (100.0)	1 (100.0)
≥ 3	–	0 (0.0)	0 (0.0)	≥ 3	–	0 (0.0)	0 (0.0)
Not assessed/not evaluable	–	0 (0.0)	0 (0.0)	Not assessed/not evaluable	–	0 (0.0)	0 (0.0)
**Sensation**	–	3	3	**Sensation**	–	1	1
0 – 2	–	3 (100.0)	3 (100.0)	0 – 2	–	1 (100.0)	1 (100.0)
≥ 3	–	0 (0.0)	0 (0.0)	≥ 3	–	0 (0.0)	0 (0.0)
Not assessed/not evaluable	–	0 (0.0)	0 (0.0)	Not assessed/not evaluable	–	0 (0.0)	0 (0.0)
**Strength**	–	3	3	**Strength**	–	1	1
0 – 2	–	3 (100.0)	3 (100.0)	0 – 2	–	1 (100.0)	1 (100.0)
≥ 3	–	0 (0.0)	0 (0.0)	≥ 3	–	0 (0.0)	0 (0.0)
Not assessed/not evaluable	–	0 (0.0)	0 (0.0)	Not assessed/not evaluable	–	0 (0.0)	0 (0.0)

**Visual fields**	–	3	3	**Visual fields**	–	1	1
0 – 2	–	3 (100.0)	3 (100.0)	0 – 2	–	1 (100.0)	1 (100.0)
≥ 3	–	0 (0.0)	0 (0.0)	≥ 3	–	0 (0.0)	0 (0.0)
Not assessed/not evaluable	–	0 (0.0)	0 (0.0)	Not assessed/not evaluable	–	0 (0.0)	0 (0.0)
**Cycle 6 Day 22**				**Cycle 13 Day 22**			
**Ataxia**	–	3	3	**Ataxia**	–	1	1
0 – 2	–	3 (100.0)	3 (100.0)	0 – 2	–	1 (100.0)	1 (100.0)
≥ 3	–	0 (0.0)	0 (0.0)	≥ 3	–	0 (0.0)	0 (0.0)
Not assessed/not evaluable	–	0 (0.0)	0 (0.0)	Not assessed/not evaluable	–	0 (0.0)	0 (0.0)
**Behaviour**	–	3	3	**Behaviour**	–	1	1
0 – 2	–	3 (100.0)	3 (100.0)	0 – 2	–	1 (100.0)	1 (100.0)
≥ 3	–	0 (0.0)	0 (0.0)	≥ 3	–	0 (0.0)	0 (0.0)
Not assessed/not evaluable	–	0 (0.0)	0 (0.0)	Not assessed/not evaluable	–	0 (0.0)	0 (0.0)
**Facial strength**	–	3	3	**Facial strength**	–	1	1
0 – 2	–	3 (100.0)	3 (100.0)	0 – 2	–	1 (100.0)	1 (100.0)
≥ 3	–	0 (0.0)	0 (0.0)	≥ 3	–	0 (0.0)	0 (0.0)
Not assessed/not evaluable	–	0 (0.0)	0 (0.0)	Not assessed/not evaluable	–	0 (0.0)	0 (0.0)
**Gait**	–	3	3	**Gait**	–	1	1
0 – 2	–	3 (100.0)	3 (100.0)	0 – 2	–	1 (100.0)	1 (100.0)
≥ 3	–	0 (0.0)	0 (0.0)	≥ 3	–	0 (0.0)	0 (0.0)
Not assessed/not evaluable	–	0 (0.0)	0 (0.0)	Not assessed/not evaluable	–	0 (0.0)	0 (0.0)
**Language**	–	3	3	**Language**	–	1	1
0 – 2	–	3 (100.0)	3 (100.0)	0 – 2	–	1 (100.0)	1 (100.0)
≥ 3	–	0 (0.0)	0 (0.0)	≥ 3	–	0 (0.0)	0 (0.0)
Not assessed/not evaluable	–	0 (0.0)	0 (0.0)	Not assessed/not evaluable	–	0 (0.0)	0 (0.0)
**Level of consciousness**	–	3	3	**Level of consciousness**	–	1	1
0 – 2	–	3 (100.0)	3 (100.0)	0 – 2	–	1 (100.0)	1 (100.0)
≥ 3	–	0 (0.0)	0 (0.0)	≥ 3	–	0 (0.0)	0 (0.0)
Not assessed/not evaluable	–	0 (0.0)	0 (0.0)	Not assessed/not evaluable	–	0 (0.0)	0 (0.0)
**Sensation**	–	3	3	**Sensation**	–	1	1
0 – 2	–	3 (100.0)	3 (100.0)	0 – 2	–	1 (100.0)	1 (100.0)
≥ 3	–	0 (0.0)	0 (0.0)	≥ 3	–	0 (0.0)	0 (0.0)
Not assessed/not evaluable	–	0 (0.0)	0 (0.0)	Not assessed/not evaluable	–	0 (0.0)	0 (0.0)
**Strength**	–	3	3	**Strength**	–	1	1

0 – 2	–	3 (100.0)	3 (100.0)	0 – 2	–	1 (100.0)	1 (100.0)
≥ 3	–	0 (0.0)	0 (0.0)	≥ 3	–	0 (0.0)	0 (0.0)
Not assessed/not evaluable	–	0 (0.0)	0 (0.0)	Not assessed/not evaluable	–	0 (0.0)	0 (0.0)
**Visual fields**	–	3	3	**Visual fields**	–	1	1
0 – 2	–	3 (100.0)	3 (100.0)	0 – 2	–	1 (100.0)	1 (100.0)
≥ 3	–	0 (0.0)	0 (0.0)	≥ 3	–	0 (0.0)	0 (0.0)
Not assessed/not evaluable	–	0 (0.0)	0 (0.0)	Not assessed/not evaluable	–	0 (0.0)	0 (0.0)
**Cycle 7 Day 1**				**Cycle 14 Day 1**			
**Ataxia**	–	3	3	**Ataxia**	–	1	1
0 – 2	–	3 (100.0)	3 (100.0)	0 – 2	–	1 (100.0)	1 (100.0)
≥ 3	–	0 (0.0)	0 (0.0)	≥ 3	–	0 (0.0)	0 (0.0)
Not assessed/not evaluable	–	0 (0.0)	0 (0.0)	Not assessed/not evaluable	–	0 (0.0)	0 (0.0)
**Behaviour**	–	3	3	**Behaviour**	–	1	1
0 – 2	–	3 (100.0)	3 (100.0)	0 – 2	–	1 (100.0)	1 (100.0)
≥ 3	–	0 (0.0)	0 (0.0)	≥ 3	–	0 (0.0)	0 (0.0)
Not assessed/not evaluable	–	0 (0.0)	0 (0.0)	Not assessed/not evaluable	–	0 (0.0)	0 (0.0)
**Facial strength**	–	3	3	**Facial strength**	–	1	1
0 – 2	–	3 (100.0)	3 (100.0)	0 – 2	–	1 (100.0)	1 (100.0)
≥ 3	–	0 (0.0)	0 (0.0)	≥ 3	–	0 (0.0)	0 (0.0)
Not assessed/not evaluable	–	0 (0.0)	0 (0.0)	Not assessed/not evaluable	–	0 (0.0)	0 (0.0)
**Gait**	–	3	3	**Gait**	–	1	1
0 – 2	–	3 (100.0)	3 (100.0)	0 – 2	–	1 (100.0)	1 (100.0)
≥ 3	–	0 (0.0)	0 (0.0)	≥ 3	–	0 (0.0)	0 (0.0)
Not assessed/not evaluable	–	0 (0.0)	0 (0.0)	Not assessed/not evaluable	–	0 (0.0)	0 (0.0)
**Language**	–	3	3	**Language**	–	1	1
0 – 2	–	3 (100.0)	3 (100.0)	0 – 2	–	1 (100.0)	1 (100.0)
≥ 3	–	0 (0.0)	0 (0.0)	≥ 3	–	0 (0.0)	0 (0.0)
Not assessed/not evaluable	–	0 (0.0)	0 (0.0)	Not assessed/not evaluable	–	0 (0.0)	0 (0.0)
**Level of consciousness**	–	3	3	**Level of consciousness**	–	1	1
0 – 2	–	3 (100.0)	3 (100.0)	0 – 2	–	1 (100.0)	1 (100.0)
≥ 3	–	0 (0.0)	0 (0.0)	≥ 3	–	0 (0.0)	0 (0.0)
Not assessed/not evaluable	–	0 (0.0)	0 (0.0)	Not assessed/not evaluable	–	0 (0.0)	0 (0.0)
**Sensation**	–	3	3	**Sensation**	–	1	1
0 – 2	–	3 (100.0)	3 (100.0)	0 – 2	–	1 (100.0)	1 (100.0)

≥ 3	–	0 (0.0)	0 (0.0)	≥ 3	–	0 (0.0)	0 (0.0)
Not assessed/not evaluable	–	0 (0.0)	0 (0.0)	Not assessed/not evaluable	–	0 (0.0)	0 (0.0)
**Strength**	–	3	3	**Strength**	–	1	1
0 – 2	–	3 (100.0)	3 (100.0)	0 – 2	–	1 (100.0)	1 (100.0)
≥ 3	–	0 (0.0)	0 (0.0)	≥ 3	–	0 (0.0)	0 (0.0)
Not assessed/not evaluable	–	0 (0.0)	0 (0.0)	Not assessed/not evaluable	–	0 (0.0)	0 (0.0)
**Visual fields**	–	3	3	**Visual fields**	–	1	1
0 – 2	–	3 (100.0)	3 (100.0)	0 – 2	–	1 (100.0)	1 (100.0)
≥ 3	–	0 (0.0)	0 (0.0)	≥ 3	–	0 (0.0)	0 (0.0)
Not assessed/not evaluable	–	0 (0.0)	0 (0.0)	Not assessed/not evaluable	–	0 (0.0)	0 (0.0)
**Cycle 7 Day 22**				**Cycle 14 Day 22**			
**Ataxia**	–	3	3	**Ataxia**	–	1	1
0 – 2	–	3 (100.0)	3 (100.0)	0 – 2	–	1 (100.0)	1 (100.0)
≥ 3	–	0 (0.0)	0 (0.0)	≥ 3	–	0 (0.0)	0 (0.0)
Not assessed/not evaluable	–	0 (0.0)	0 (0.0)	Not assessed/not evaluable	–	0 (0.0)	0 (0.0)
**Behaviour**	–	3	3	**Behaviour**	–	1	1
0 – 2	–	3 (100.0)	3 (100.0)	0 – 2	–	1 (100.0)	1 (100.0)
≥ 3	–	0 (0.0)	0 (0.0)	≥ 3	–	0 (0.0)	0 (0.0)
Not assessed/not evaluable	–	0 (0.0)	0 (0.0)	Not assessed/not evaluable	–	0 (0.0)	0 (0.0)
**Facial strength**	–	3	3	**Facial strength**	–	1	1
0 – 2	–	3 (100.0)	3 (100.0)	0 – 2	–	1 (100.0)	1 (100.0)
≥ 3	–	0 (0.0)	0 (0.0)	≥ 3	–	0 (0.0)	0 (0.0)
Not assessed/not evaluable	–	0 (0.0)	0 (0.0)	Not assessed/not evaluable	–	0 (0.0)	0 (0.0)
**Gait**	–	3	3	**Gait**	–	1	1
0 – 2	–	3 (100.0)	3 (100.0)	0 – 2	–	1 (100.0)	1 (100.0)
≥ 3	–	0 (0.0)	0 (0.0)	≥ 3	–	0 (0.0)	0 (0.0)
Not assessed/not evaluable	–	0 (0.0)	0 (0.0)	Not assessed/not evaluable	–	0 (0.0)	0 (0.0)
**Language**	–	3	3	**Language**	–	1	1
0 – 2	–	3 (100.0)	3 (100.0)	0 – 2	–	1 (100.0)	1 (100.0)
≥ 3	–	0 (0.0)	0 (0.0)	≥ 3	–	0 (0.0)	0 (0.0)
Not assessed/not evaluable	–	0 (0.0)	0 (0.0)	Not assessed/not evaluable	–	0 (0.0)	0 (0.0)
**Level of consciousness**	–	3	3	**Level of consciousness**	–	1	1
0 – 2	–	3 (100.0)	3 (100.0)	0 – 2	–	1 (100.0)	1 (100.0)
≥ 3	–	0 (0.0)	0 (0.0)	≥ 3	–	0 (0.0)	0 (0.0)

Not assessed/not evaluable	–	0 (0.0)	0 (0.0)
**Sensation**	–	3	3
0 – 2	–	3 (100.0)	3 (100.0)
≥ 3	–	0 (0.0)	0 (0.0)
Not assessed/not evaluable	–	0 (0.0)	0 (0.0)
**Strength**	–	3	3
0 – 2	–	3 (100.0)	3 (100.0)
≥ 3	–	0 (0.0)	0 (0.0)
Not assessed/not evaluable	–	0 (0.0)	0 (0.0)
**Visual fields**	–	3	3
0 – 2	–	3 (100.0)	3 (100.0)
≥ 3	–	0 (0.0)	0 (0.0)
Not assessed/not evaluable	–	0 (0.0)	0 (0.0)
Not assessed/not evaluable	–	0 (0.0)	0 (0.0)
**Sensation**	–	1	1
0 – 2	–	1 (100.0)	1 (100.0)
≥ 3	–	0 (0.0)	0 (0.0)
Not assessed/not evaluable	–	0 (0.0)	0 (0.0)
**Strength**	–	1	1
0 – 2	–	1 (100.0)	1 (100.0)
≥ 3	–	0 (0.0)	0 (0.0)
Not assessed/not evaluable	–	0 (0.0)	0 (0.0)
**Visual fields**	–	1	1
0 – 2	–	1 (100.0)	1 (100.0)
≥ 3	–	0 (0.0)	0 (0.0)
Not assessed/not evaluable	–	0 (0.0)	0 (0.0)
**End of Study**	–		
**Ataxia**	2	7	9
0 – 2	1 (50.0)	7 (100.0)	8 (88.9)
≥ 3	0 (0.0)	0 (0.0)	0 (0.0)
Not assessed/not evaluable	1 (50.0)	0 (0.0)	1 (11.1)
**Behaviour**	2	7	9
0 – 2	2 (100.0)	7 (100.0)	9 (100.0)
≥ 3	0 (0.0)	0 (0.0)	0 (0.0)
Not assessed/not evaluable	0 (0.0)	0 (0.0)	0 (0.0)
**Facial strength**	2	7	9
0 – 2	2 (100.0)	7 (100.0)	9 (100.0)
≥ 3	0 (0.0)	0 (0.0)	0 (0.0)
Not assessed/not evaluable	0 (0.0)	0 (0.0)	0 (0.0)
**Gait**	2	7	9
0 – 2	2 (100.0)	5 (71.4)	7 (77.8)
≥ 3	0 (0.0)	2 (28.6)	2 (22.2)
Not assessed/not evaluable	0 (0.0)	0 (0.0)	0 (0.0)
**Language**	2	7	9
0 – 2	2 (100.0)	7 (100.0)	9 (100.0)
≥ 3	0 (0.0)	0 (0.0)	0 (0.0)
Not assessed/not evaluable	0 (0.0)	0 (0.0)	0 (0.0)

	**Level of consciousness**	2	7	9
	0 – 2	2 (100.0)	7 (100.0)	9 (100.0)
	≥ 3	0 (0.0)	0 (0.0)	0 (0.0)
	Not assessed/not evaluable	0 (0.0)	0 (0.0)	0 (0.0)
	**Sensation**	2	7	9
	0 – 2	2 (100.0)	7 (100.0)	9 (100.0)
	≥ 3	0 (0.0)	0 (0.0)	0 (0.0)
	Not assessed/not evaluable	0 (0.0)	0 (0.0)	0 (0.0)
	**Strength**	2	7	9
	0 – 2	2 (100.0)	7 (100.0)	9 (100.0)
	≥ 3	0 (0.0)	0 (0.0)	0 (0.0)
	Not assessed/not evaluable	0 (0.0)	0 (0.0)	0 (0.0)
	**Visual fields**	2	7	9
	0 – 2	1 (50.0)	5 (71.4)	6 (66.7)
	≥ 3	1 (50.0)	1 (14.3)	2 (22.2)
	Not assessed/not evaluable	0 (0.0)	1 (14.3)	1 (11.1)

† NANO assessment only for patients with brain tumour, including glioblastoma, of whom received any amount of auceliciclib and completed a post-baseline disease response assessment. NANO, Neurologic Assessment in Neuro-Oncology; *n*, sample size; NA, not available.

Supplementary Table S7. Plasma pharmacokinetic profile of auceliciclib following single and repeated oral administrations

Mean (% CV)	50 mg od	100 mg od	150 mg od	250 mg od	350 mg od	175 mg b.i.d.	250 mg b.i.d.	500 mg b.i.d.
**Single dosing**	*n* = 1	*n* = 1	*n* = 4	*n* = 3	*n* = 3	*n* = 3	*n* = 3	*n* = 2
**C_{max} (ng/ml)**	27.2	188	103 (61.6)	139 (92.2)	163 (43.8)	225 (44.3)	235 (60.2)	232 (6.10)
**t_{max} (h)**†	6.00	2.78	5.80 (2.98 – 8.02)	3.83 (2.00 – 4.02)	2.97 (2.97 – 23.9)	3.93 (3.02 – 24.28)	7.77 (3.83 – 24.1)	24.00 (23.17 – 24.83)
**$AUC_{0\text{-}tlast}$ (h × ng/ml)**	747	2970	2230 (45.8)	1750 (57.2)	3230 (3.41)	3050 (41.6)	4180 (56.6)	4000 (3.52)
**$AUC_{0\text{-}12}$ (h × ng/ml)**	257	1080	763 (59.0)	806 (71.6)	1180 (37.0)	1480 (66.3)	1730 (51.2)	1490 (15.9)
**$t_{1/2}$ (h)**§	26.7	32.7	25.4 (24.2)	21.9 (42.3)	20.0‡ (19.3)	NE	NE	NE
**CL/F (l/h)**§	47.1	22.4	54.9 (48.4)	133 (43.3)	86.9‡ (11.2)	NE	NE	NE
**V_z/F (l)**§	1810	1060	1970 (43.1)	4470 (78.3)	2480‡ (8.21)	NE	NE	NE
**Repeated dosing**	*n* = 1	*n* = 1	*n* = 2	*n* = 3	*n* = 3	*n* = 2	*n* = 3	*n* = 2
**C_{max} (ng/ml)**	120	251	266 (31.9)	174 (69.0)	388 (72.9)	330 (13.1)	266 (40.5)	383 (31.8)
**t_{max} (h)**†	3.07	7.60	3.48 (2.92 – 4.03)	3.78 (2.02 – 4.00)	3.92 (2.02 – 4.00)	4.03 (3.85 – 4.20)	3.88 (2.95 – 5.62)	5.03 (4.05 – 6.00)
**$AUC_{0\text{-}tlast}$ (h × ng/ml)**	1510	7920	5350 (27.7)	2800 (46.0)	3890 (18.8)	5740 (15.0)	5000 (53.2)	6760 (24.6)
**$AUC_{0\text{-}12}$ (h × ng/ml)**	911	2760	2190 (26.4)	1220 (54.8)	3290 (80.7)	3040 (1.85)	2570 (48.9)	3770 (28.3)
**$t_{1/2}$ (h)**§	17.2	26.3	22.6 (8.81)	21.5 (18.6)	21.0‡ (25.5)	NE	NE	NE
**CL/F (l/h)**§	33.6	19.9	42.8 (26.2)	148 (43.8)	98.1 (60.3)	57.5 (1.85)	112 (40.4)	138 (28.3)
**V_z/F (l)**§	836	758	1380 (17.6)	4790 (60.1)	3850‡ (4.52)	NE	NE	NE

†Data reported in median (range). ‡Data for 350 mg od based on two patients only (third patient had insufficient data for parameter estimation). §NE due to no valid extrapolation found (i.e. insufficient data point, high variability or inappropriate sampling schedule). $AUC_{0\text{-}12}$, area under the concentration time curve from 0 to 12 hours post dose; $AUC_{0\text{-}tlast}$, area under the concentration time curve from zero to the last quantifiable time-point; b.i.d., twice daily; C_{max}, maximum observed concentration; CL/F, apparent clearance; CV, coefficient of variation; *n*, sample size; NE, not estimable; od, once daily; $t_{1/2}$, apparent terminal elimination half-life; t_{max}, time to C_{max}; V_z/F, apparent volume of distribution.

Supplementary Table S8. Plasma pharmacokinetic profile of single and repeated dosing of auceliciclib in combination with 100 mg TMZ

Mean (% CV)	100 mg od	150 mg od	100 mg b.i.d.	150 mg b.i.d.	300 mg b.i.d.	500 mg b.i.d.
**Single dosing**	*n* = 1	*n* = 1	*n* = 5	*n* = 3	*n* = 3	*n* = 4
**C_{max} (ng/ml)**	12.3	47.0	31.4 (33.5)	35.7 (24.4)	113 (71.5)	239 (57.5)
**t_{max} (h)†**	4.00	4.07	6.00 (3.20 – 23.82)	22.53 (4.02 – 23.75)	3.87 (2.97 – 23.73)	24.26 (3.87 – 24.68)
**$AUC_{0\text{-}tlast}$ (h × ng/ml)**	236	692	446 (56.0)	655 (28.6)	1850 (78.6)	3900 (62.0)
**$AUC_{0\text{-}12}$ (h × ng/ml)**	114	418	242‡ (47.4)	286 (38.3)	815 (82.7)	1580 (79.1)
**$t_{1/2}$ (h)$^{\#}$**	NE	NE	NE	NE	NE	NE
**CL/F (l/h)$^{\#}$**	NE	NE	NE	NE	NE	NE
**V_z/F (l)$^{\#}$**	NE	NE	NE	NE	NE	NE
**Repeated dosing§**	–	–	–	–	*n* = 3	*n* = 2
**C_{max} (ng/ml)**	–	–	–	–	160 (63.9)	292 (83.8)
**t_{max} (h)†**	–	–	–	–	3.93 (3.92 – 4.00)	4.89 (3.93 – 5.85)
**$AUC_{0\text{-}tlast}$ (h × ng/ml)**	–	–	–	–	1020 (64.8)	2210 (108)
**$AUC_{0\text{-}12}$ (h × ng/ml)**	–	–	–	–	1440 (74.4)	4780¶
**$t_{1/2}$ (h)$^{\#}$**	–	–	–	–	NE	NE
**CL/F (l/h)$^{\#}$**	–	–	–	–	289 (59.2)	105¶
**V_z/F (l)$^{\#}$**	–	–	–	–	NE	NE

†Data reported in median (range). ‡Data for 100 mg b.i.d. based on four patients only (fifth patient had insufficient data for parameter estimation). §Repeated-dose plasma samples were not collected for pharmacokinetic analysis in the 100 mg od, 150 mg od, 100 mg b.i.d., and 150 mg b.i.d. cohorts before protocol amendment. ¶Data for 500 mg b.i.d. based on one patient only (second patient had insufficient data for parameter estimation). $^{\#}$NE due to no valid extrapolation found (i.e. insufficient data point, high variability or inappropriate sampling schedule). $AUC_{0\text{-}12}$, area under the concentration time curve from 0 to 12 hours post dose; $AUC_{0\text{-}tlast}$, area under the concentration time curve from zero to the last quantifiable time-point; b.i.d., twice daily; C_{max}, maximum observed concentration; CL/F, apparent clearance; CV, coefficient of variation; *n*, sample size; NE, not estimable; od, once daily; $t_{1/2}$, apparent terminal elimination half-life; t_{max}, time to C_{max}; V_z/F, apparent volume of distribution.

Supplementary Table S9. Summary of TEAEs based on dose cohorts

	Phase I							
**Number of patients with any (*n* (%)[†])**	**50 mg od (*n* = 1)**	**100 mg od (*n* = 1)**	**150 mg od (*n* = 4)**	**250 mg od (*n* = 3)**	**350 mg od (*n* = 3)**	**175 mg b.i.d. (*n* = 3)**	**250 mg b.i.d. (*n* = 3)**	**500 mg b.i.d. (*n* = 2)**
Serious TEAEs	0 (0.0)	0 (0.0)	0 (0.0)	0 (0.0)	0 (0.0)	1 (33.3)	1 (33.3)	0 (0.0)
TEAEs leading to study withdrawal	0 (0.0)	0 (0.0)	0 (0.0)	0 (0.0)	0 (0.0)	0 (0.0)	0 (0.0)	0 (0.0)
TEAEs leading to discontinuation of auceliciclib	0 (0.0)	0 (0.0)	0 (0.0)	0 (0.0)	0 (0.0)	0 (0.0)	1 (33.3)	1 (50.0)
TEAEs leading to dose interruption of auceliciclib	0 (0.0)	1 (100.0)	0 (0.0)	0 (0.0)	1 (33.3)	1 (33.3)	1 (33.3)	0 (0.0)
TEAEs leading to dose reduction of auceliciclib	0 (0.0)	0 (0.0)	0 (0.0)	0 (0.0)	0 (0.0)	0 (0.0)	0 (0.0)	0 (0.0)

	Phase IIa						
**Number of patients with any (*n* (%)[†])**	**100 mg od (*n* = 1)**	**150 mg od (*n* = 1)**	**100 mg b.i.d. (*n* = 5)**	**150 mg b.i.d. (*n* = 3)**	**300 mg b.i.d. (*n* = 3)**	**500 mg b.i.d. (*n* = 4)**	**All (*n* = 37)**
Serious TEAEs	0 (0.0)	1 (100.0)	1 (20.0)	0 (0.0)	0 (0.0)	1 (25.0)	5 (13.5)
TEAEs leading to study withdrawal	0 (0.0)	0 (0.0)	0 (0.0)	0 (0.0)	0 (0.0)	1 (25.0)	1 (2.7)
TEAEs leading to discontinuation of auceliciclib	0 (0.0)	0 (0.0)	0 (0.0)	0 (0.0)	0 (0.0)	2 (50.0)	4 (10.8)
TEAEs leading to discontinuation of TMZ	0 (0.0)	0 (0.0)	0 (0.0)	0 (0.0)	0 (0.0)	2 (50.0)	2 (5.4)
TEAEs leading to dose interruption of auceliciclib	0 (0.0)	0 (0.0)	1 (20.0)	0 (0.0)	0 (0.0)	0 (0.0)	5 (13.5)
TEAEs leading to dose interruption of TMZ	0 (0.0)	0 (0.0)	1 (20.0)	0 (0.0)	0 (0.0)	1 (25.0)	2 (5.4)
TEAEs leading to dose reduction of auceliciclib	0 (0.0)	0 (0.0)	0 (0.0)	0 (0.0)	0 (0.0)	1 (25.0)	1 (2.7)
TEAEs leading to dose reduction of TMZ	0 (0.0)	0 (0.0)	0 (0.0)	0 (0.0)	0 (0.0)	0 (0.0)	0 (0.0)

[†]Percentages are calculated (the denominator used for the calculation) based on the number of patients of each treatment group. The number of patients is counted once for the highest relationship to auceliciclib for a given adverse event in patient count (*n*). b.i.d., twice daily; od, once daily; TEAE, treatment-emergent adverse event; TMZ, temozolomide.

Supplementary Table S10. Summary of MGMT promoter methylation and IDH status in patients with high grade glioma (phase IIa)

Dose cohort	Sex (Age)	Tumour type[†]	IDH status	MGMT promoter methylation status
100 mg od	M (61)	High-grade glioma (GBM)	Wild-type	Not reported
150 mg od	M (62)	High-grade glioma (GBM)	Wild-type	Not reported
100 mg b.i.d.	M (56)	High-grade glioma (GBM)	Wild-type	Not reported
	M (67)	High-grade glioma (GBM)	Wild-type	Unmethylated
	F (38)	High-grade glioma (GBM)	Wild-type	Unmethylated
	M (48)	High-grade glioma (GBM)	Wild-type	Methylated
	M (65)	High-grade glioma (GBM)	Wild-type	Unmethylated
150 mg b.i.d.	M (53)	High-grade glioma (GBM)	Wild-type	Not reported
	M (66)	High-grade glioma (GBM)	Wild-type	Methylated
	F (40)	High-grade glioma (unspecified)	Mutant	Methylated
300 mg b.i.d.	M (46)	High-grade glioma (anaplastic astrocytoma)	Mutant	Not reported
	M (48)	High-grade glioma (GBM)	Wild-type	Unmethylated
	M (70)	High-grade glioma (GBM)	Wild-type	Unmethylated
500 mg b.i.d.	M (61)	High-grade glioma (GBM)	Wild-type	Unmethylated
	F (44)	High-grade glioma (GBM)	Wild-type	Methylated
	M (58)	High-grade glioma (GBM)	Wild-type	Not reported
	M (70)	High-grade glioma (GBM)	Wild-type	Methylated

[†]All cases were reviewed per the 2021 WHO classification, which restricts the diagnosis of GBM to IDH-wild-type tumours; cases not meeting these criteria or not re-evaluated were reclassified as high-grade glioma, with specific tumour type noted where available. b.i.d., twice daily; F, female; GBM, glioblastoma; IDH, isocitrate dehydrogenase; M, male; MGMT, O^6-methylguanine-DNA-methyltransferase; od, once daily.

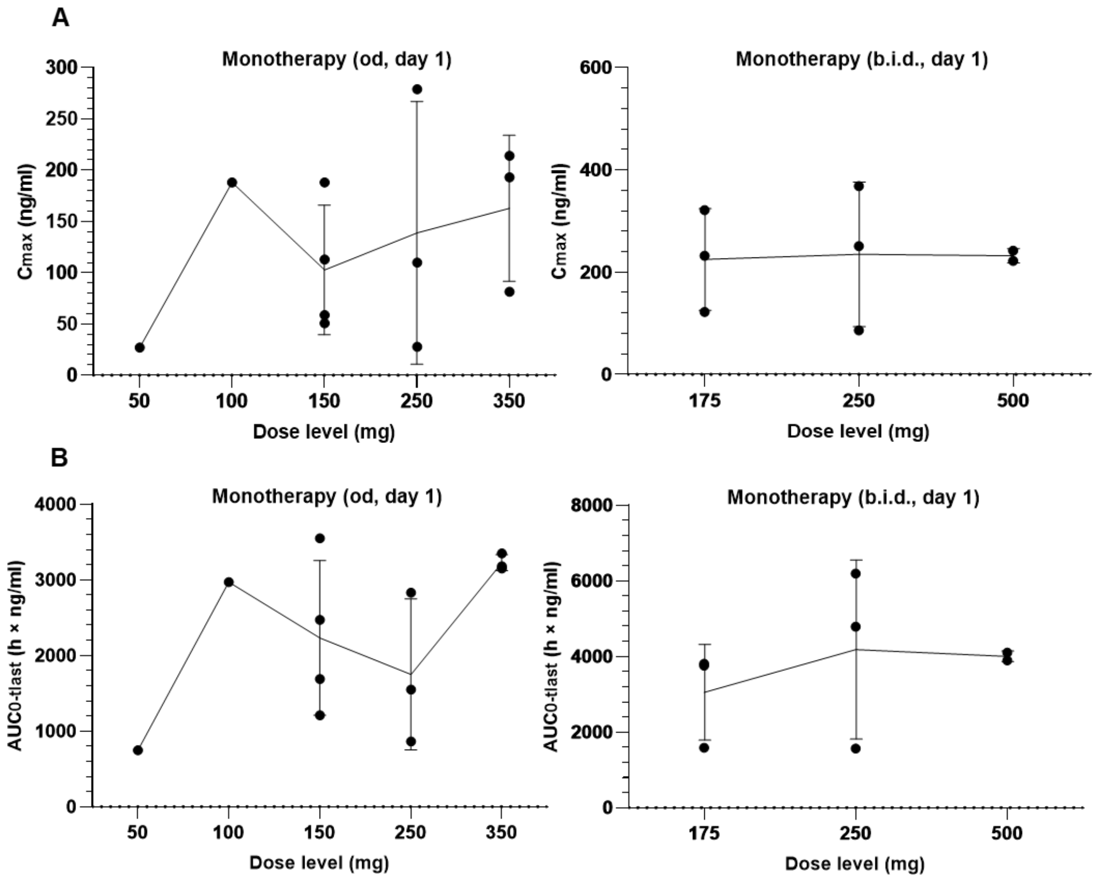

Supplementary Figure S1. Mean plasma pharmacokinetic parameters of auceliciclib monotherapy across various cohorts. (A) C_{max} and (B) $AUC_{0\text{-}tlast}$ of auceliciclib following total daily dosing administered either od or b.i.d. on cycle 1 day 1, across a dose range of 50 to 1000 mg. Each dot represents individual pharmacokinetic data; lines connect mean values at each dose level, with error bars indicating standard deviations.

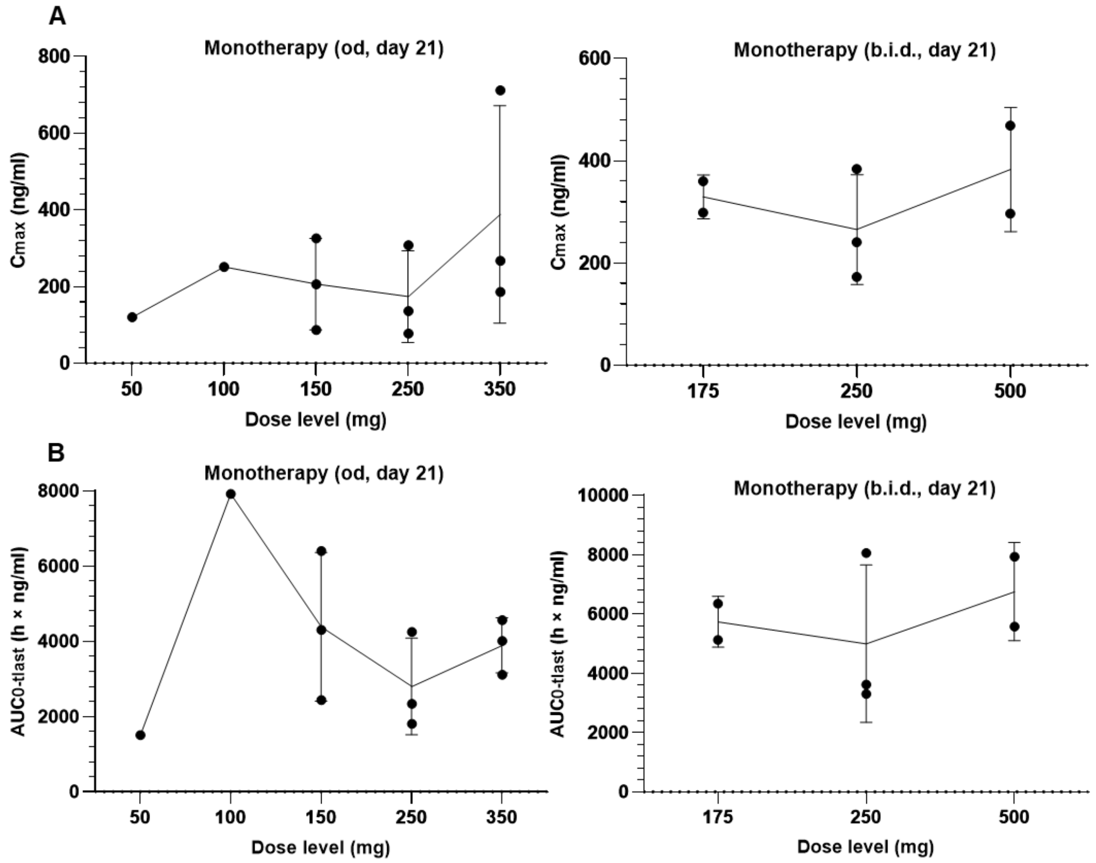

Supplementary Figure S2. Mean plasma pharmacokinetic parameters of auceliciclib monotherapy across various cohorts. (A) C_{max} and (B) $AUC_{0\text{-tlast}}$ of auceliciclib following total daily dosing administered either od or b.i.d. on cycle 1 day 21, across a dose range of 50 to 1000 mg. Each dot represents individual pharmacokinetic data; lines connect mean values at each dose level, with error bars indicating standard deviations. One patient data from the 150 mg od cohort was collected on day 15 instead of day 21.

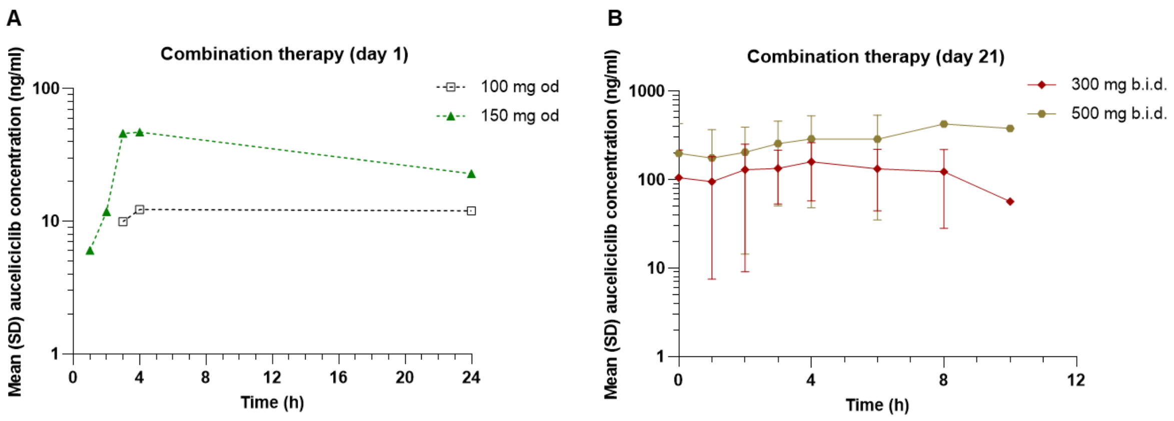

Supplementary Figure S3. Plasma concentration profiles of auceliciclib following oral administration. Mean (± standard deviation) plasma concentration-time curves of auceliciclib in combination with 100 mg TMZ, with total daily doses ranging from 100 to 1000 mg on cycle 1 (A) day 1 and (B) day 21.

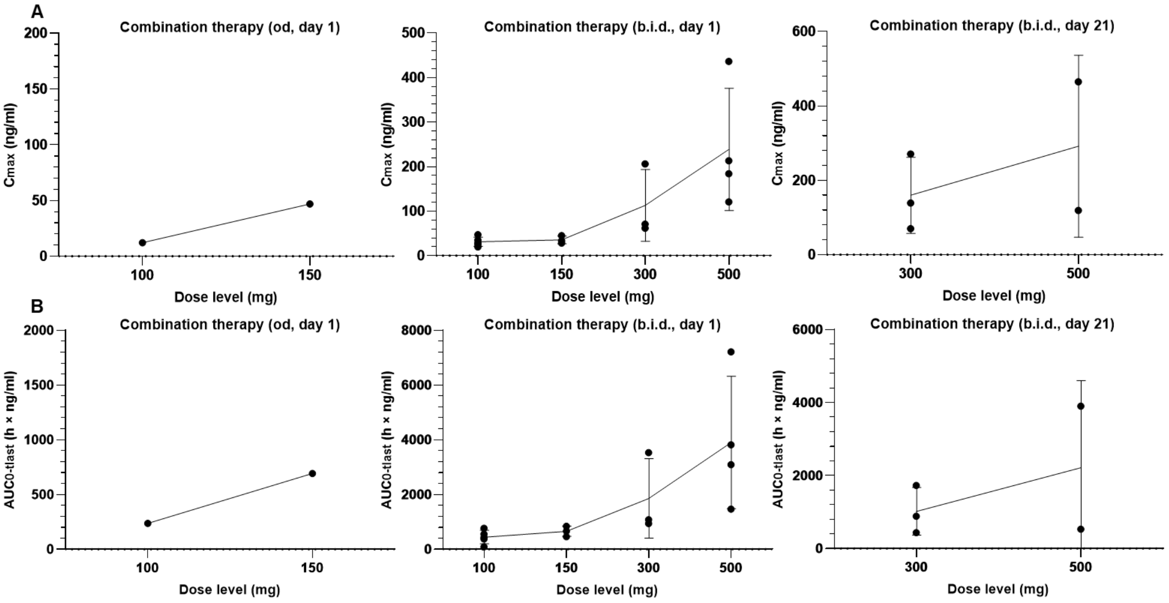

Supplementary Figure S4. Mean plasma pharmacokinetic parameters of auceliciclib in combination therapy with TMZ across various cohorts. (A) C_{max} and (B) $AUC_{0\text{-}tlast}$ of auceliciclib following total daily dosing administered either od or b.i.d. on cycle 1 day 1 and cycle 1 day 21, across a dose range of 100 to 1000 mg, with 100 mg TMZ od. Each dot represents individual pharmacokinetic data; lines connect mean values at each dose level, with error bars indicating standard deviations.

References

1. US Food and Drug Administration. Guidance for industry: estimating the maximum safe starting dose in initial clinical trials for therapeutics in adult healthy volunteers. 2005. https://www.fda.gov/media/72309/download. Accessed 26 Dec, 2020.
2. International Council for Harmonisation of Technical Requirements for Pharmaceuticals for Human Use. Nonclinical evaluation for anticancer pharmaceuticals. ICH Harmonised Tripartite Guideline S9. Current Step 4 version dated 29 October 2009. https://database.ich.org/sites/default/files/S9_Guideline.pdf. Accessed 26 Dec, 2020.
3. Simon R, Freidlin B, Rubinstein L, Arbuck SG, Collins J, Christian MC. Accelerated titration designs for phase I clinical trials in oncology. *J Natl Cancer Inst.* 1997;89(15):1138-1147.
